# The role of the tyrosine kinase Wzc (Sll0923) and the phosphatase Wzb (Slr0328) in the production of extracellular polymeric substances (EPS) by *Synechocystis* PCC 6803

**DOI:** 10.1002/mbo3.753

**Published:** 2019-01-23

**Authors:** Sara B. Pereira, Marina Santos, José P. Leite, Carlos Flores, Carina Eisfeld, Zsófia Büttel, Rita Mota, Federico Rossi, Roberto De Philippis, Luís Gales, João H. Morais‐Cabral, Paula Tamagnini

**Affiliations:** ^1^ i3S ‐ Instituto de Investigação e Inovação em Saúde Universidade do Porto Porto Portugal; ^2^ IBMC ‐ Instituto de Biologia Molecular e Celular Universidade do Porto Porto Portugal; ^3^ ICBAS – Instituto de Ciências Biomédicas Abel Salazar Porto Portugal; ^4^ Department of Agrifood Production and Environmental Sciences University of Florence Florence Italy; ^5^ Faculdade de Ciências, Departamento de Biologia Universidade do Porto Porto Portugal; ^6^ Present address: Department of Water Management Delft University of Technology Delft The Netherlands; ^7^ Present address: Molecular Microbiology, Groningen Biomolecular Sciences and Biotechnology Institute University of Groningen Groningen The Netherlands

**Keywords:** cyanobacteria, extracellular polymeric substances, *Synechocystis*, Wzb, Wzc

## Abstract

Many cyanobacteria produce extracellular polymeric substances (EPS) mainly composed of heteropolysaccharides with unique characteristics that make them suitable for biotechnological applications. However, manipulation/optimization of EPS biosynthesis/characteristics is hindered by a poor understanding of the production pathways and the differences between bacterial species. In this work, genes putatively related to different pathways of cyanobacterial EPS polymerization, assembly, and export were targeted for deletion or truncation in the unicellular *Synechocystis* sp. PCC 6803. No evident phenotypic changes were observed for some mutants in genes occurring in multiple copies in *Synechocystis* genome, namely ∆*wzy* (∆*sll0737*), ∆*wzx* (∆*sll5049*), ∆*kpsM* (∆*slr2107*), and ∆*kpsM*∆*wzy* (∆*slr2107*∆*sll0737*), strongly suggesting functional redundancy. In contrast, Δ*wzc* (Δ*sll0923*) and Δ*wzb* (Δ*slr0328*) influenced both the amount and composition of the EPS, establishing that Wzc participates in the production of capsular (CPS) and released (RPS) polysaccharides, and Wzb affects RPS production. The structure of Wzb was solved (2.28 Å), revealing structural differences relative to other phosphatases involved in EPS production and suggesting a different substrate recognition mechanism. In addition, Wzc showed the ATPase and autokinase activities typical of bacterial tyrosine kinases. Most importantly, Wzb was able to dephosphorylate Wzc in vitro, suggesting that tyrosine phosphorylation/dephosphorylation plays a role in cyanobacterial EPS production.

## 1 INTRODUCTION

Polysaccharide‐based biopolymers can provide a diverse and powerful platform to deliver a wide range of biological and functional properties to the industrial toolbox. However, and despite the overwhelming diversity of polymers synthesized by microorganisms, bacterial polysaccharides are still underrepresented in the market (Roca, Alves, Freitas, & Reis, 2015). Most cyanobacterial strains produce extracellular polymeric substances (EPS), mainly composed of heteropolysaccharides that can remain attached to the cell surface (CPS—capsular polysaccharides) or be released into the environment (RPS—released polysaccharides) (Pereira et al., 2009; Rossi & De Philippis, 2016). The distinctive features of the cyanobacterial EPS, including their strong anionic nature, presence of sulfate groups, high variety of possible structural conformations, and amphiphilic behavior, make these polymers suitable for biotechnological and biomedical applications such as flocculating, gelifying, emulsifying, or suspending agents, rheology modifiers, therapeutic or drug delivery agents (Leite et al., 2017; Pereira et al., 2009). The faster growth and easier genetic manipulation of cyanobacteria compared to algae and plants, and the low‐cost biomass production (owing to their photosynthetic metabolism) are additional competitive advantages for the implementation of cyanobacteria as cost‐effective cell factories for the production of these biopolymers. For this purpose, a deeper knowledge on the cyanobacterial EPS biosynthetic pathways is required to optimize the production and engineer structural and compositional variants tailored for a given application.

The mechanisms involved in EPS production seem to be relatively conserved throughout bacteria, with the polymerization, assembly, and export of the polymers usually following one of three main mechanisms, namely the Wzy‐, ABC transporter‐, or synthase‐dependent pathways (Schmid, Sieber, & Rehm, 2015). However, in a phylum‐wide analysis, we showed that most cyanobacteria harbor gene‐encoding proteins related to the three pathways but often not the complete set defining a single pathway (Pereira, Mota, Vieira, Vieira, & Tamagnini, 2015), implying a more complex scenario than that observed for other bacteria. This complexity is also evident in the physical organization of the genes, with multiple copies scattered throughout the genomes, either isolated or in small clusters (Pereira et al., 2015, 2009). In particular, this analysis revealed that *Synechocystis* sp. PCC 6803 (hereafter *Synechocystis*) possesses the genes related to the Wzy‐ and/or ABC transporter‐dependent pathways, whereas those related to the synthase‐dependent pathway are mostly absent (Pereira et al., 2015). Mutational analyses showed that the ORFs *slr0977*,* slr0982*,* sll0574,* and *sll0575* putatively encoding ABC transporter components (TCDB: 3.A.1.103.5) operate in *Synechocystis*’ EPS production (Fisher, Allen, Luo, & Curtiss, 2013). Likewise, *sll0923* and *sll1581*, encoding homologs of the polysaccharide copolymerase (PCP) Wzc (TCDB: 8.A.3) and outer membrane polysaccharide export (OPX) Wza (TCDB: 1.B.18) of the Wzy‐dependent pathway, were also shown to be involved (Jittawuttipoka et al., 2013).

In bacteria, Wzc and Wza form a complex that spans the periplasmic space and promotes the export of EPS polymer. Wzc undergoes a phospholyation/dephosphorylation cycle that affects its oligomerization state and is dependent on the phosphatase activity of another protein, Wzb (Cuthbertson, Mainprize, Naismith, & Whitfield, 2009). The *Synechocystis*’ Wzc homolog, Sll0923, possesses a C‐terminal cytoplasmic domain harboring the Walker A, A’, and B ATP‐binding motifs and Y‐rich region present in other Wzc proteins and characteristic of bacterial tyrosine kinases (BY‐kinases) (Mijakovic, Grangeasse, & Turgay, 2016; Morona, Purins, Tocilj, Matte, & Cygler, 2009; Pereira et al., 2015; Pereira, Mota, Santos, Philippis, & Tamagnini, 2013; Standish & Morona, 2014). In addition, Sll0328 was identified as a low molecular weight protein tyrosine phosphatase (LMW‐PTP; EC: 3.1.3.48) (Mukhopadhyay & Kennelly, 2011) and Wzb homolog. Altogether, this raises the possibility that EPS production is at least partially controlled by a tyrosine phosphoregulatory mechanism, similar to that observed in other organisms (Grangeasse, Cozzone, Deutscher, & Mijakovic, 2007; Mijakovic et al., 2016; Standish & Morona, 2014).

In this work, aiming at elucidating the process of EPS production in cyanobacteria, we generated an array of *Synechocystis*’ mutants and characterized them in terms of growth, amount of EPS produced, and polymer composition. The results obtained demonstrate that Wzc (Sll0923) and Wzb (Sll0328) are involved in the production of EPS, influencing both the amount and the composition of polymer(s). The absence of both Wzc and Wzb seems to redirect RPS production toward an alternative route. We clarified the roles of both proteins through biochemical and structural analysis, providing the first insights into the molecular mechanisms of EPS production in this cyanobacterium.

## 2 MATERIALS AND METHODS

### 2.1 Organisms and growth conditions

The cyanobacterium *Synechocystis* sp. PCC 6803 (Pasteur Culture Collection) and mutant strains (Supporting Information Table S1) were cultured in BG11 medium (Stanier, Kunisawa, Mandel, & Cohen‐Bazire, 1971) at 30°C, under a 12‐hr light (50 µmol photons m^−1^ s^−2^)/12‐hr dark regime and orbital agitation (150 rpm). For solid medium, BG11 was supplemented with 1.5% agar noble (Difco), 0.3% sodium thiosulfate, and 10 mM TES‐KOH buffer (pH 8.2). For the selection and maintenance of mutants, BG11 medium was supplemented with kanamycin (Km, up to 700 µg/ml), spectinomycin (Sm, up to 50 µg/ml), and/or chloramphenicol (Cm, up to 25 µg/ml). The *Escherichia coli* strains used were cultured at 37°C in LB medium (Bertani, 1951) supplemented with Amp (100 µg/ml), Km (25 µg/ml), and/or Cm (25 µg/ml).

### 2.2 Cyanobacterial DNA extraction and recovery

Cyanobacterial genomic DNA was extracted using the Maxwell^®^ 16 System (Promega) except to use in Southern blot, for which the phenol/chloroform method previously described (Tamagnini, Troshina, Oxelfelt, Salema, & Lindblad, 1997) was preferred. Agarose gel electrophoresis was performed by standard protocols (Sambrook & Russell, 2001), and the DNA fragments were isolated from gels, enzymatic, or PCR reactions using the NZYGelpure purification kit (NZYTech).

### 2.3 Plasmid construction for *Synechocystis* transformation

Plasmid pDsll0923::Km^r^ was kindly provided by F. Chauvat (Jittawuttipoka et al., 2013). The *Synechocystis* chromosomal regions flanking *wzy* (*sll0737*), *wzx* (*sll5049*), *kpsM* (*slr2107*), *wzb* (*slr0328*), or the last 78 bp of *wzc* (*sll0923*) were amplified by PCR using the specific oligonucleotide primers (Supporting Information Appendix S1 and Supporting Information Table S2). An overlapping region containing an *Xma*I restriction site was included in primers 5I and 3I for cloning purposes. For each gene, the purified PCR fragments were fused by “overlap‐PCR” (Supporting Information Appendix S1). The resulting products were purified and cloned into the vector pGEM‐T^®^ Easy (Promega), originating pGDsll0737, pGDsll5049, pGDslr2107, pGDslr0328, and pGDsll0923_Trunc_. A selection cassette containing the *nptII* gene (conferring resistance to neomycin and kanamycin) was amplified from pKm.1 using the primer pair Km.KmScFwd/KmRev (Pinto et al., 2015) (Supporting Information Appendix S1 and Supporting Information Table S2) and digested with *Xma*I (Thermo Scientific). Subsequently, the purified selection cassette was cloned in the *Xma*I restriction site of the plasmids using the T4 DNA ligase (Thermo Scientific) to form pGDsll0737.Km, pGDsll5049.Km, pGDslr2107.Km, pGDslr0328.Km, or pGDsll0923Trunc.Km, respectively. The cassette containing the *aadA* gene (conferring resistance to streptomycin and spectinomycin) was obtained by digesting the plasmid pSEVA481 (Silva‐Rocha et al., 2013) with *Psh*AI and *Swa*I, and the cassette was cloned in the *Xma*I/*Sma*I site of pGDslr0328 and pGDsll0727 to form plasmids pGDslr0328.Sm and pGDsll0727.Sm.

For mutants’ complementation, the shuttle vector pSEVA351 (Silva‐Rocha et al., 2013) was used. A fragment covering *wzc* and its native promoter (P*_wzc_*) and RBS (−230 to +123, with +1 corresponding to transcriptional start site (Kopf et al., 2014)) was amplified using primer pair sll0923_compF1/sll0923_compR1 (Supporting Information Appendix S1 and Supporting Information Table S2). The purified product was cloned in pGEM‐T^®^ Easy after A‐tailing and subsequently digested with *Xba*I and *Spe*I. The resultant DNA fragment was cloned into pSEVA351 previously digested with *Xba*I and *Spe*I, originating plasmid pS351sll0923. To obtain plasmid pS351sll0923_Trunc,_ a similar procedure was used but the PCR was performed using primer pair sll0923_compF1/Sll0923.RTrunc (Supporting Information Appendix S1 and Supporting Information Table S2), and the digested fragment was cloned into the *Xba*I site of pSEVA351. Since *wzb* is part of a four gene operon (slr0326‐slr0329) (Kopf et al., 2014), a different approach was used. The P*_rnpB_* promoter (Huang, Camsund, Lindblad, & Heidorn, 2010) was obtained by digesting the BioBrick vector pSB1C3 with *Xba*I and *Spe*I and subsequently cloned in pSEVA351 previously digested with the same enzymes, originating plasmid pS351P*_rnpB_*. *wzb* was amplified using primer pair slr0328_compF/slr0328_compR, incorporating the synthetic RBS BBa_B0030 (Supporting Information Appendix S1 and Supporting Information Table S2). The purified product was cloned in pGEM‐T^®^ Easy after A‐tailing, digested with *Xba*I and *Spe*I and subsequently cloned in the *Spe*I site of pS351P*_rnpB_* generating plasmid pS351slr0328.

All constructs were verified by sequencing (StabVida) before transformation of *Synechocystis*.

### 2.4 Generation of *Synechocystis* mutants

*Synechocystis* was transformed with integrative plasmids using the procedure described previously (Williams, 1988). Briefly, *Synechocystis* cultures were grown until OD_730_ around 0.5, cells were harvested by centrifugation and resuspended in one‐tenth volume of BG11. One hundred microliter of cells were incubated with 6–20 µg/ml plasmid DNA for 5 hr before spread onto Immobilon™‐NC membranes (0.45 µm pore size, Millipore) resting on solid BG11 plates at 30°C under continuous light. After 24 hr, the membranes were transferred to selective plates containing 10 µg/ml of kanamycin or 2.5 µg/ml of spectinomycin. Transformants were observed after 1–2 weeks. For complete segregation, colonies were grown at increasing antibiotic concentrations. Nonintegrative plasmids were transformed into *Synechocystis* by electroporation, as previously described (Chiaramonte, Giacometti, & Bergantino, 1999; Ludwig, Heimbucher, Gregor, Czerny, & Schmetterer, 2008). In this case, cells were washed with HEPES buffer, 1 mM pH 7.5. Afterwards, cells were resuspended in 1 ml HEPES and 60 μl were mixed with 1 μg of DNA and electroporated with a Bio‐Rad Gene Pulser^TM^, at a capacitor of 25 μF. The resistor used was 400 Ω for time constant of 9 ms with an electric field of 12 kV/cm. Immediately after the electric pulse, the cells were resuspended in 1 ml BG11 and spread onto the Immobilon™‐NC membranes as described above. After 24 hr, the membranes were transferred to selective plates containing 10 µg/ml of chloramphenicol before grown at increasing antibiotic concentrations.

### 2.5 Southern blots

Southern blots were performed using genomic DNA of the wild type and mutants digested with *Bam*HI (∆*wzy* and ∆*kpsM*∆*wzy*), *Eco*RI (∆*kpsM* and ∆*kpsM*∆*wzy*), *Ava*II (∆*wzx*), *Nco*I (∆*wzc, wzc*_Trunc_ and ∆*wzc*∆*wzb*), and/or *Mfe*I (∆*wzb* and ∆*wzc*∆*wzb*) (Thermo Scientific). The DNA fragments were separated by electrophoresis on a 1% agarose gel and blotted onto Amersham Hybond^TM^‐N membrane (GE Healthcare). Probes were amplified by PCR and labeled using the primers indicated in Supporting Information Table S2 and DIG DNA labeling kit (Roche Diagnostics GmbH) according to the manufacturer's instructions. Hybridization was done overnight at 60°C (∆*wzc*, and ∆*wzc*∆*wzb*) or 65ºC (∆*wzy*, ∆*wzx*, ∆*kpsM*, ∆*kpsM*∆*wzy*, ∆*wzb,* ∆*wzc*∆*wzb,* and *wzc*_Trunc_), and digoxigenin‐labeled probes were detected by chemiluminescence using CPD‐star (Roche Diagnostics GmbH) in a Chemi DocTM XRS+Imager (Bio‐Rad).

### 2.6 Transcription analysis

For RNA extraction, 100 ml of culture of *Synechocystis* sp. PCC 6803 wild type, ∆*wzy* (*∆sll0737*), ∆*wzx* (*∆sll5049*), or ∆*kpsM* (*∆slr2107*) mutants (at OD_730nm_ ≈ 1) were collected 6 hr into the light phase. RNA extraction, quantification, quality/integrity assessment were carried out as previously described (Pinto, Pacheco, Ferreira, Moradas‐Ferreira, & Tamagnini, 2012). The absence of genomic DNA contamination was checked by PCR using primers for *rnpB* amplification and the following profile: 5 min at 95°C followed by 30 cycles of 30 s at 95°C, 30 s at 56°C, and 30 s at 72°C, and a final extension at 72°C for 7 min. After synthesis with random primers as described previously (Pinto et al., 2012), cDNAs were used as template in PCR amplifications with the oligonucleotide primers listed in Supporting Information Table S2. The PCR profile used was: 5 min at 95°C followed by 30 cycles of 40 s at 95°C, 40 s at 54°C, and 40 s at 72°C, and a final extension at 72°C for 7 min. A control PCR was performed for *rnpB* amplification as described above. Band intensities were estimated by ImageJ software (Schneider, Rasband, & Eliceiri, 2012).

### 2.7 Growth measurements

Growth measurements were performed by monitoring the Optical Density (OD) at 730 nm (Anderson & McIntosh, 1991) using a Shimadzu UVmini‐1240 (Shimadzu Corporation) and determining the chlorophyll *a* content as described previously (Meeks & Castenholz, 1971). Data were statistically analyzed as described below.

### 2.8 Analysis of total carbohydrates, RPS and CPS

Total carbohydrates and RPS contents were determined as described previously (Mota et al., 2013). For the quantification of CPS, 5 ml of dialyzed cultures was centrifuged at 3,857 *g* for 15 min at room temperature, resuspended in water, and boiled for 15 min at 10°C to detach the CPS from the cells’ surface. After new centrifugation as described above, CPS were quantified from the supernatants using the phenol‐sulfuric acid method (Dubois, Gilles, Hamilton, Rebers, & Smith, 1956). Total carbohydrate, RPS, and CPS were expressed as mg per L of culture or normalized by optical density. Data were statistically analyzed as described below.

To determine the RPS’ monosaccharidic composition, dialyzed cultures were centrifuged at 3,857 *g* for 10 min at room temperature to remove the cells and the supernatant was further centrifuged at 75,000 *g* for 1 hr 30 min at 15°C to remove LPS prior to lyophilization. 2–5 mg of isolated RPS was hydrolyzed with 1 ml of 2 M trifluoroacetic acid (TFA) at 120ºC for 1 hr. Samples were analyzed by ion exchange chromatography using a Dionex ICS‐2500 ion chromatograph with an ED50 pulsed amperometric detector using a gold working electrode (Dionex) and a Carbopac PA1 column (Dionex). The eluents used were (A) MilliQ‐grade water, (B) 0.185 M sodium hydroxide solution, and (C) 0.488 M sodium acetate solution. The gradient consisted of a first stage with 84% solution A, 15% solution B, and 1% solution C (for 7 min); a second stage with 50% solution B and 50% solution C (for 9 min); and a final stage with 84% solution A, 15% solution B, and 1% solution C (for 14 min). The flow rate was 1 ml/min.

### 2.9 LPS extraction and analysis

LPS extraction was performed according to (Simkovsky et al., 2012) with some modifications. Briefly, 25 ml of samples was collected by centrifugation at 3,857 *g* for 15 min at room temperature, washed once in BG11, and incubated on ice for 30 min in stripping buffer (sucrose 15% (w/v), Tris‐HCl 50 mM, and Na_2_EDTA 25 mM). After centrifugation at 3,802 *g* for 10 min at 4°C, the supernatant was transferred to a new tube and centrifuged at 16,170 *g* for 2 min at 4°C to remove minor cell contaminants. The supernatant was collected and centrifuged at 75,000 *g* for 90 min at 18ºC. The pellet was resuspended in 100 µl of 10 mM Tris‐HCl, pH 8.0. Protease digested LPS samples were analyzed in a 12% SDS‐PAGE gel (Bio‐Rad Laboratories) and stained using Pro‐Q^®^ Emerald 300 Lipopolysaccharide Gel Stain Kit (Molecular Probes, Inc.) according to the manufacturer's instructions.

### 2.10 Transmission electron microscopy (TEM)

Cells were fixed before centrifugation and processed as described previously (Seabra, Santos, Pereira, Moradas‐Ferreira, & Tamagnini, 2009), except that samples were embedded in EMBed‐812 resin and sections were examined using a JEM‐1400Plus (Jeol Ltd., Inc.). Negative staining was performed on cells mounted on formvar/carbon film‐coated mesh nickel grids (Electron Microscopy Sciences) with 1% Ruthenium Red.

### 2.11 Expression and purification of Wzb and Wzc

To overexpress N‐terminal his‐tagged Wzb (His6‐Wzb, 18.9 kDa) and Wzc (His6‐Wzc, 84.9 kDa), the genes were amplified from *Synechocystis* genomic DNA using the oligonucleotide pairs Ovslr0328F/Ovslr0328R or OvSll0923F/OvSll0923R, respectively (Supporting Information Table S2). The products obtained were digested with *Shp*I/*Pst*I or *Bam*HI/*Pst*I, respectively, and cloned into pQE‐30 (QIAGEN). After confirming by sequencing (StabVida) that no mutations had been introduced, the constructs were introduced into M15 (pREP4) cells (QIAGEN). Transformed *E. coli* cells were grown in LB medium supplemented with 100 µg/ml of ampicillin and 25 µg/ml of kanamycin, at 37°C, until an optical density at 600 nm of 0.6 for Wzb or 0.7–0.9 for Wzc. To express His6‐Wzb, cells were induced for 1 hr at 37°C with 0.5 mM IPTG and lysed in a Branson sonifier 250 (Duty cycle 50%, output 5, 3 × 10 s) in Wzb lysis buffer (50 mM HEPES pH 8.0, 300 mM NaCl, 20 mM imidazole, 0.5% Triton X‐100, 0.2 mg/ml lysozyme, 10 µg/ml DNase, 1 mM MgCl_2,_ and 1 mM phenylmethylsulfonyl fluoride—PMSF). After centrifugation at 35,000 *g* for 30 min at 4°C, His6‐Wzb was purified using HisTrap affinity columns (GE Healthcare). Samples were loaded in Wzb‐binding buffer (50 mM HEPES pH 8.0, 300 mM NaCl, 20 mM imidazole, 0.5% Triton X‐100), and bound proteins were eluted using a step gradient in which imidazole increased up to 500 mM. Samples were concentrated and diafiltered with HEPES 50 mM pH 8.0 or further purified by size exclusion chromatography (SEC), using a Superpose12 10/300 column (GE healthcare) and Wzb SEC buffer (50 mM HEPES pH 8.0 and 100 mM NaCl). For His6‐Wzc, cultures were subjected to a 30‐min cold shock before induction with 0.5 mM IPTG and incubation at 20°C overnight. Cells were disrupted using a French Press (Thermo Electron Corporation) at 30 Kpsi in Wzc lysis buffer (50 mM HEPES pH 8.0, 100 mM NaCl, 0.2 mg/ml lysozyme, 10 µg/ml DNase, 1 mM MgCl_2,_ and 1 mM PMSF). After centrifugation at 30,000 *g* for 30 min at 4°C to remove cell debris, the membrane fraction was collected at 200,000 *g* for 1 hr 10 min at 4ºC and resuspended in Wzc buffer A (50 mM HEPES pH 8.0, 100 mM NaCl). Triton X‐100 was added to a final concentration of 10%, and samples were incubated for 1 hr at 4ºC with orbital shaking to maximize protein solubilization. His6‐Wzc was purified as described above, except that the composition of the Wzc binding buffer was 50 mM HEPES pH 8.0, 0.10 M NaCl, 20 mM imidazole, and 0.2% Triton X‐100. Pooled fractions containing His6‐Wzc were concentrated and dialyzed (Dialysis Membrane, 25 kDa MWCO,Spectra/Por) against buffer A supplemented with 1 mM *n*‐Dodecyl‐b‐maltoside (DDM) ON at 4°C. Protein was further purified by SEC using a HiPrep 16/60 Sephacryl S‐300 High Resolution column (GE healthcare) with Wzc SEC buffer (50 mM HEPES pH 8.0, 100 mM NaCl, 1 mM DDM, and 2.5% glycerol). The concentrations of the purified His‐tagged protein solutions were determined by BCA colorimetric assay (Thermo Scientific) using bovine serum albumin as standard.

### 2.12 Wzb phosphatase activity assay

The phosphatase activity of His6‐Wzb was determined by continuously monitoring, at 405 nm, the formation of *p*‐nitrophenol (pNP) from *p*‐nitrophenyl phosphate (pNPP), at 30°C, in a Shimadzu UV‐2401 PC (Shimadzu Corporation). 1 ml of reaction mixture contained 100 mM sodium citrate buffer, pH 6.5, 1 mM EDTA, 0.1% (vol/vol) β‐mercaptoethanol, and 10 mM of pNPP. Reactions were initiated by adding 0.2 or 0.5 µg of purified His6‐Wzb. The concentration of pNP formed was estimated using a molar extinction coefficient of 18,000 M^−1^ cm^−1^ (Ferreira et al., 2007; Preneta et al., 2002). Data were statistically analyzed as described below.

### 2.13 Wzb crystallization, data collection, and processing

For the initial screening, His6‐Wzb aliquots at 10 mg/ml were used. The screening was performed in 24‐well sitting‐drop vapor diffusion plates at 20°C with several commercially available kits. A single hit was obtained with MembFac (Hampton Research) condition #22. After optimization with solutions with varying pH and precipitant concentrations, the best diffracting crystals appeared between pH 6.2 and 7.0 and 1 M of ammonium sulfate. Crystals were subjected to a glycerol gradient up to 30% prior to being flash frozen in liquid nitrogen. A data set of a single diffracting crystal to 2.28 Å was determined at the ID23–2 beamline of the European Synchrotron Radiation Facility (*λ* = 0.873 Å; Grenoble, France). Diffraction images were processed with the XDS Program Package (Kabsch, 1993), and the diffraction intensities converted to structure factors in the CCP4 format (Bailey, 1994). A random 5% sample of the reflection data was flagged for R‐free calculations (Brunger, 1992) during model building and refinement. A summary of the data collection and refinement statistics is presented in Table 2. Molecular replacement phases were generated with PhaserMR (McCoy et al., 2007), using as initial model the protein tyrosine phosphatase from *Entamoeba histolytica* (PDB entry 3IDO; (Linford et al., 2014)). The final models were obtained after further cycles of refinement and manual model building, carried out with PHENIX (Adams et al., 2010) and Coot (Emsley, Lohkamp, Scott, & Cowtan, 2010), respectively. Protein structure figures were generated with PyMol (Schrödinger, 2010).

### 2.14 Phylogenetic analysis of Wzb

*Synechocystis*’ Wzb sequence was used as query in blast searches against the PDB database (Jan 2017; https://www.rcsb.org/) (Berman et al., 2000) to retrieve homologs with 3D crystal structures available, using a significance cutoff of e‐05. Sequences were aligned in MEGA6 (Tamura et al., 2013) using the ClustalW algorithm, and the phylogenetic tree was constructed in the same software by maximum likelihood, using the Jones‐Taylor‐Thornton (JTT) substitution model and a bootstrap of 500. A three‐dimensional protein structure alignment was performed using representative LMW‐PTP sequences from the eukaryote *E. histolytica* (PDB: 3ido; UniProt:C4LSE7), the Gram‐negative bacteria *E. coli* (PDB: 2wja; UniProt:Q9X4B8) and the Gram‐positive bacteria *Staphylococcus aureus* (PDB: 3rof; UniProt: P0C5D2), and the root mean square deviation (RMSD) was calculated as previously described (Krissinel & Henrick, 2004).

### 2.15 Dephosphorylation of Wzc by Wzb

The dephosphorylation reaction was monitored by Western immunoblot analysis. For that, 1 µg of both His6‐Wzc and His6‐Wzb was incubated in 20 µl of reaction buffer (100 mM sodium citrate, pH 6.5, and 1 mM EDTA) at 30°C for 0, 1, 2, 4, 6, 12, or 24 hr. Reactions were terminated by adding SDS‐PAGE sample buffer. Samples were heated at 95°C for 5 min, separated on 4%–15% SDS‐PAGE gels (Bio‐Rad), and transferred onto nitrocellulose membranes as previously described (Leitao, Oxelfelt, Oliveira, Moradas‐Ferreira, & Tamagnini, 2005). Membranes were probed with either monoclonal anti‐phosphotyrosine antibody (PT‐66; Sigma) diluted 1:2,000, or 6x‐His Epitope Tag Antibody (Thermo Scientific) diluted 1:1,000. Membranes were then incubated with goat anti‐mouse IgG‐HRP (Santa Cruz Biotechnology) at a dilution of 1:5,000. Immunodetection was performed using the ECL^TM^ Western blotting detection reagents (GE healthcare) or the WesternBright^TM^ Quantum (Advansta) and a Chemi DocTM XRS+Imager (Bio‐Rad). The relative signal intensity of the bands obtained by immunodetection was quantified using the Image Lab software (Bio‐Rad). Data were statistically analyzed as described below.

### 2.16 Wzc ATPase activity

The ATPase activity of His6‐Wzc was determined using the ENLITEN^®^ ATP Assay System Bioluminescence Detection Kit for ATP Measurement (Promega). Samples were incubated in 25 mM Tris‐HCl pH 7.0, 1 mM DTT, and 5 mM MgCl_2_ at 30ºC for 45 min before reading luminescence using a Synergy^TM^ 2 Multi‐Mode Microplate Reader and Gen5^TM^ software (BioTek) with an integration time of 10 s. When necessary, His6‐Wzc and His6‐Wzc were inactivated by incubation at 95ºC for 5 min before adding ATP. Data were statistically analyzed as described below.

### 2.17 Wzc autokinase activity

The autokinase activity of His6‐Wzc was evaluated using everted membrane vesicles overexpressing the protein. For that, *E. coli* M15 (pREP4) cells harboring pQE‐30::His6‐Wzc or empty pQE‐30 were grown as described above. Everted membrane vesicles were prepared from the *E. coli* cells following the protocol previously described (Rosen, 1986), except that buffer B contained 150 mM KCl instead of choline chloride and 1 mM PMSF. Isolated vesicles were incubated in dephosphorylation buffer with purified His6‐Wzb using a total protein ratio of 20:1 for 0 or 6 hr. Subsequently, the dephosphorylation buffer and excess of His6‐Wzb were removed by dialyzing against kinase buffer (100 mM Tris‐HCl pH 8.0, 200 mM KCl, 1 mM MgCl_2_) using membranes with a cutoff of 25 kDa (Spectra/Por^®^; Spectrum Labs) for a minimum of 16 hr at 4ºC. Dephosphorylated vesicles were incubated in kinase buffer supplemented or not with 200 μM ATP for 0 hr, 10 min, 30 min, or 6 hr. The kinase reaction was monitored by Western immunoblot, and statistical analyses were performed as described below.

### 2.18 Analysis of Wzc phosphorylation

Protein bands were excised from stained gels, and samples were processed for mass spectrometry analysis as previously described (Gomes et al., 2013; Osório & Reis, 2013). Briefly, protein spots were sequentially washed with ultrapure water, 50% acetonitrile in 50 mM ammonium bicarbonate (ABC) followed by dehydration with 100% acetonitrile. Afterward, protein spots were reduced with 25 mM dithiothreitol in 50 mM ABC, at 56ºC for 20 min and alkylated with 55 mM iodoacetamide in 50 mM ABC, for 20 min at room temperature in the dark, followed by the above described washing/dehydration procedures. In‐gel protein enzymatic digestion was performed using trypsin in the presence of 0.01% surfactant (Promega) for 3 hr at 37ºC. Resulting peptides were extracted from gel plugs with 2.5% TFA for 15 min at 1,400 rpm (Thermomixer, Eppendorf), dried under vacuum (SpeedVac, Thermo Scientific), and resuspended in 0.1% TFA.

Protein identification was performed by MALDI mass spectrometry (4800 Plus MALDI TOF/TOF Analyzer; SCIEX). Protein digests were purified by reversed‐phase C18 chromatography (ZipTips, Millipore) following manufacturer's instructions and eluted in the MALDI sample plate using the MALDI matrix alpha‐Cyano‐4‐hydroxycinnamic acid (CHCA) as elution solution at 8 mg/ml in 50% ACN, 0.1% TFA, 6 mM ammonium phosphate. Peptide mass spectra were acquired in reflector positive mode in the mass range of *m*/*z* 700–5,000. Relevant peptide peaks were selected for MS/MS sequencing. Proteins were identified by Peptide Mass Fingerprint (PMF) approach with the Mascot software (v2.5.1, Matrix Science) using the UniProt protein sequence database for the taxonomic selection *Synechocystis* (2017_01 release). MS/MS phosphopeptide sequencing followed by Mascot analysis was performed for phosphorylation site determination. The protein search settings were cysteine carbamidomethylation (constant modification), methionine oxidation, and tyrosine phosphorylation (variable modifications), up to two missed trypsin cleavages, and maximum error tolerance of 10 ppm (MS)/0.5 Da (MS/MS). Protein scores >51 were considered significant (*p* < 0.05).

### 2.19 Statistical analysis

Data were statistically analyzed in GraphPad Prism v7 (GraphPad Software) using a one‐way analysis of variance (ANOVA), followed by Tukey's multiple comparisons.

## 3 RESULTS

### 3.1 Wzc and Wzb play a role in *Synechocystis*’ EPS production

**Figure 1:**
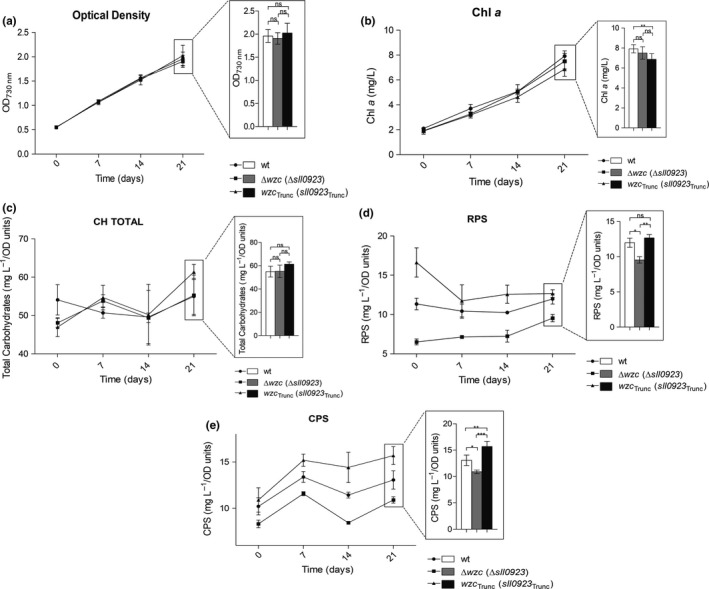
Characterization of *Synechocystis* sp. PCC 6803 wild type and Δ*wzc* and *wzc*_Trunc_ mutants in terms of growth ([a] optical density at *λ* = 730 nm [OD_730nm_] and [b] µg of chlorophyll *a* per ml of culture [Chl *a*]), and production of (c) total carbohydrates, (d) released polysaccharides (RPS), and (e) capsular polysaccharides (CPS) expressed as mg per OD730_nm_ units. Experiments were performed in triplicate. Data are means ± *SD*. Statistical analysis performed using one‐way analysis of variance (ANOVA), followed by Tukey's multiple comparisons, is presented for the last time point. Significant differences are identified: *(*p* ≤ 0.05), **(*p* ≤ 0.01), ***(*p* ≤ 0.001). Ns: no significant differences

**Figure 2:**
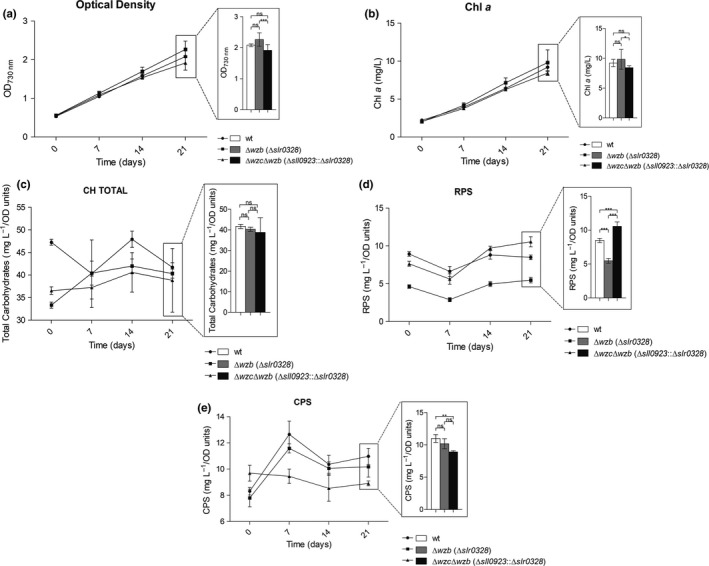
Characterization of *Synechocystis* sp. PCC 6803 wild type and Δ*wzb* and Δ*wzc*Δ*wzb* in terms of growth ([a] optical density at *λ* = 730 nm [OD_730nm_] and [b] µg of chlorophyll *a* per ml of culture [Chl *a*]), and production of (c) total carbohydrates, (d) released polysaccharides (RPS), and (e) capsular polysaccharides (CPS) expressed as mg per OD730_nm_ units. Experiments were performed in triplicate. Data are means ± *SD*. Statistical analysis performed using one‐way analysis of variance (ANOVA), followed by Tukey's multiple comparisons, is presented for the last time point. Significant differences are identified: *(*p* ≤ 0.05), **(*p* ≤ 0.01), ***(*p* ≤ 0.001). Ns: no significant differences

To unveil the key players in cyanobacterial EPS biosynthesis, we have used *Synechocystis* sp. PCC 6803 and deleted gene‐encoding proteins putatively involved in the Wzy‐ and ABC transporter‐dependent pathways. For this purpose, the genes were partially replaced with an antibiotic resistance cassette using double homologous recombination. The first fully segregated mutants obtained, namely ∆*wzy* (∆*sll0737*—encoding the polymerase), ∆*wzx* (∆*sll5049*—flippase), ∆*kpsM* (∆*slr2107*—ABC transporter component), and ∆*kpsM*∆*wzy* (∆*slr2107*∆*sll0737*), did not show any obvious phenotype in terms of growth, total carbohydrates, RPS, and CPS content (Supporting Information Figures S1–S5). We also confirmed by RT‐PCR that all the putative copies of *wzy* (*sll0737*,* slr0728*,* slr1515*,* sll5074,* and *slr1074*), *wzx* (*sll5049*,* slr0488*,* slr0896,* and *slr1543*), and *kpsM* (*slr2107*,* slr0977,* and *sll0564*) (see Pereira et al., 2015; Kopf et al., 2014) were transcribed under standard laboratory conditions (Supporting Information Figure S6). Subsequently, we generated ∆*wzc* (∆*sll0923*), ∆*wzb* (∆*slr0328*), ∆*wzc*∆*wzb* (∆*sll0923*∆*slr0328*), and *wzc*_Trunc_ mutants. The last strain possesses a truncated Wzc, lacking the last 25 amino acids that constitute the C‐terminal Y‐rich region, where autophosphorylation and dephosphorylation by Wzb are expected to occur. Although a ∆*wzc* mutant strain was already described, we generated a ∆*wzc* in our *Synechocystis* strain using the construct kindly provided by Jittawuttipoka et al. (2013) to avoid phenotypic changes arising from the use of different *Synechocystis* substrains (Trautmann, Voß, Wilde, Al‐Babili, & Hess, 2012). In all cases, the mutant strains were fully segregated (Supporting Information FigureS7) and did not display significant growth differences compared to the wild type (Figures 1 and 2), indicating that the targeted genes are not essential in standard laboratory conditions. With the exception of *wzc*_Trunc_, the chlorophyll *a* content showed a linear correlation with OD values, proving to be a good estimation of cell density/number (Figures 1 and 2). The amount of total carbohydrates, RPS, and CPS produced per liter of culture was measured and normalized by the OD value and chlorophyll *a* content. These two approaches lead to congruent results and; therefore, only the production per OD value is shown (Figures 1c,d,e and 2c,d,e). Statistical analyses are presented for the last time point, as the differences accumulate with the increase of the cell density of the cultures. The four mutants showed different amounts of CPS and/or RPS compared to the wild type, even if none of the mutations abolished CPS and/or RPS production. ∆*wzc* showed an approximately 20% and 17% decrease in the amount of RPS (Figure 1d) and CPS (Figure 1e), respectively, confirming the role of Wzc in the production of these polymers (Jittawuttipoka et al., 2013). These authors reported a higher decrease in CPS and RPS production (about 50%). The difference to the amounts reported here is likely related to the *Synechocystis* genetic background used to generate the mutants (Trautmann et al., 2012), the growth conditions (e.g. cultivation of cells in medium with Na_2_CO_3_ by Jittawuttipoka et al., 2013), and the experimental design (e.g. time course of the experiment and normalization of the data by amount of protein by Jittawuttipoka et al., 2013). *∆wzb* exhibited 35% less RPS (Figure 2d) but no significant changes in the amount of CPS could be observed (Figure 2e), indicating that Wzb is only involved in RPS production. The double mutant ∆*wzc*∆*wzb* exhibited 18% decrease in CPS (Figure 2e) and a 23% increase of RPS (Figure 2d). The decrease of CPS displayed by the double mutant and the single mutant ∆*wzc* is consistent with the hypothesis that Wzb does not play a role in the regulation of CPS production. In contrast, the higher amount of RPS produced by the double mutant suggests that, in the absence of both proteins, the RPS production is likely to be diverted to an alternative route. Interestingly, the *wzc*_Trunc_ mutant produced the same amount of RPS and 19% more CPS compared to the wild type (Figure 1d,e), suggesting that the C‐terminal Y‐rich region of this protein is only functionally relevant in the regulation of CPS production. The apparently contradictory outcomes of the *∆wzb* and *wzc*_Trunc_ mutants have two possible explanations: (a) Wzb is not involved in the dephosphorylation of the C‐terminal Y‐rich region of Wzc; or (b) the absence of Wzb affects the phosphorylation/dephosphorylation cycles of Wzc, altering RPS production, and this effect is not replicated when the Y‐rich region of Wzc is truncated. To ensure that the observed phenotypes were not due to polar effects, the deletion mutants were complemented by the addition of *wzb* or *wzc* in *trans* restoring EPS production. This was done using the replicative vector pSEVA351 (Silva‐Rocha et al., 2013) harboring the native *wzc* or the *wzb*.

Despite the changes observed for RPS and CPS in the mutants, the amount of total carbohydrates did not vary significantly (Figures 1c and 2c), consistent with an intracellular accumulation of the polysaccharides.

It has been previously shown for other bacteria that the assembly of EPS and other surface polysaccharides, such as the O‐antigen of LPS and S‐layer glycans, follows similar mechanisms that may be functionally connected (Babu et al., 2011; Ristl et al., 2011; Simkovsky et al., 2012; Whitfield & Trent, 2014). However, for *Synechocystis*, no obvious differences were observed for the LPS profile and the cells’ surface ultrastructure comparing the wild type and the mutants (Supporting Information Figures S8 and S9). The monosaccharidic composition of the RPS produced by the different strains was also evaluated. A considerable increase in the percentage of rhamnose was observed in all mutants (Table 1). In addition, the RPS produced by *∆wzb* are composed by a smaller number of different monosaccharides. These results highlight that the differences induced by these mutations are not limited to the amount of the polymer but extend to their composition.

**Table 1:** Monosaccharidic composition of the released polysaccharides extracted from *Synechocystis* sp. PCC 6803 wild type, Δ*wzc*, Δ*wzb,* ∆*wzc*∆*wzb,* and *wzc*_Trunc_

Monosaccharide[Link]	Wild type		Δ*wzc*		Δ*wzb*		Δ*wzc*Δ*wzb*		*wzc*_Trunc_	
	Mean[Link]	*SD* (*n* = 3)	Mean[Link]	*SD* (*n* = 3)	Mean[Link]	*SD* (*n* = 3)	Mean[Link]	*SD* (*n* = 3)	Mean[Link]	*SD* (*n* = 3
Glucose	17.50	3.21	6.85	0.82	5.20	1.30	9.98	0.31	10.38	1.78
Mannose	10.17	2.66	7.19	1.95	2.52	1.02	7.13	2.80	5.06	0.18
Galactose	11.90	0.99	4.30	1.89	1.16	0.27	3.55	3.26	3.58	0.93
Fructose	np		np		np		Tr[Link]		2.36	0.55
Fucose	8.23	0.70	5.14	1.18	1.84	0.25	4.64	1.76	3.60	0.13
Ribose	4.23	1.84	Tr[Link]		np		Tr[Link]		Tr[Link]	
Rhamnose	6.19	2.55	50.51	8.47	88.60	3.34	48.32	11.47	57.81	5.30
Xylose	2.52	0.59	2.15	0.30	Tr[Link]		2.10	0.45	Tr[Link]	
Arabinose	7.74	5.17	6.93	2.78	Tr[Link]		4.61	6.19	3.58	1.11
Glucuronic acid	np		np		np		7.38	2.64	Tr[Link]	
Galacturonic acid	12.37	2.85	2.02	1.05	np		np		2.15	0.67
Glucosamine	7.46	1.70	1.87	0.67	np		Tr[Link]		2.19	0.41
Galactosamine	15.14	2.20	13.36	1.56	np		10.74	1.78	7.31	2.84

Notes

Differences discussed in the text are highlighted in gray.

Np: not present.

Mean expressed in Mol % of each monosaccharide. Sums may not be exactly 100% due to rounding.

Tr: traces <1%.

### 3.2 Wzb has a classical LMW‐PTP conformation

To better define the functional role of *Synechocystis*’ Wzb in the production of EPS, we overexpressed this protein in *E. coli* and performed its purification. The presence of recombinant protein was confirmed by SDS‐PAGE/Western blot (Supporting Information Figure S10).

**Figure 3:**
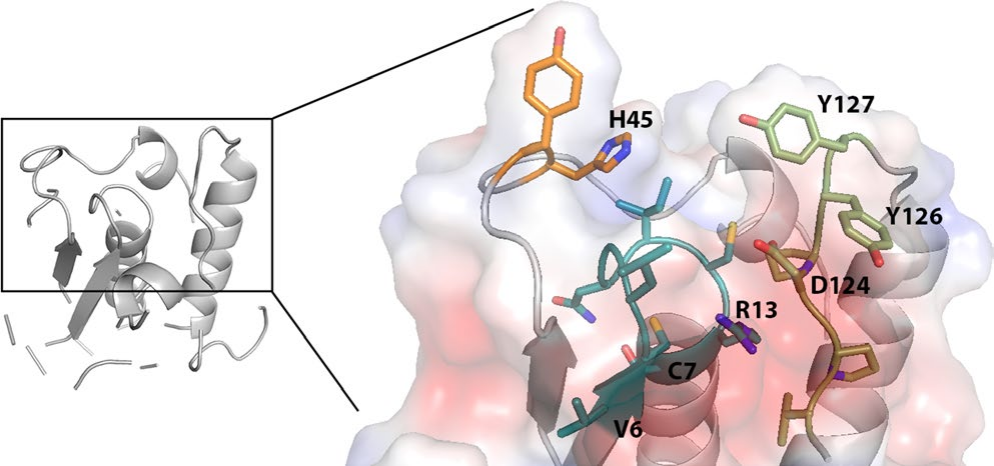
Structure of Wzb from *Synechocystis* sp. PCC 6803. A close view of the active site with surface representation is shown. The protein backbone is represented in gray; the active site residues are in stick representation, with oxygen in red, nitrogen in blue, and sulfur in yellow; the PTP, DPYY, and histidine 45‐containing loops are represented in teal, light green, and orange, respectively. Relevant residues are labeled: valine (V) 6, cysteine (C) 7, arginine (R) 13, histidine (H) 45, aspartate (D) 124, tyrosine (Y) 126, and tyrosine (Y) 127. Representations made with Pymol (Schrödinger, 2010)

The Wzb protein was crystallized and analyzed through X‐ray diffraction. Data collection and refinement statistics are shown in Table 2. One monomer was present in the asymmetric unit, with the crystal lattice belonging to the P3_1_2 1 space group. *Synechocystis*’ Wzb displays the structure of a low molecular weight protein tyrosine phosphatase (LMW‐PTP) (Figure 3). The PTPases signature motif, the PTP loop, with the characteristic sequence C(X)_5_R(S/T) (cysteine 7 to serine 14) is located between the C‐terminus of the β1 strand and the N‐terminus of the α1 helix, at the N‐terminus side of the protein (Zhang, 1998). As in other LMW‐PTP proteins, the PTP loop is preceded by a valine residue (V6) (Kolmodin & Aqvist, 2001; Mukhopadhyay & Kennelly, 2011). Opposite and topping this loop, there is the DPYY loop, which contains the functionally essential aspartate residue (D124). Together, the DPYY and PTP loops constitute the enzyme´s active site (Standish & Morona, 2014; Zhang, 1998). The tyrosine residues of the DPYY loop form one of the walls of the active site pocket, with a histidine (H45) delineating the other side of the substrate entry site.

**Table 2:** Crystallographic data collection, processing, and structure refinement statistics for Wzb

Data collection	
Space group	P3_1_2 1
Unit cell dimensions	
*a* (Å)	73.41
*b* (Å)	73.41
*c* (Å)	68.07
*α* = *β* (◦)	90
*γ* (◦)	120
Resolution range (Å)	46.46–2.28 (2.36–2.28)
No. of unique reflections	10,021 (959)
Multiplicity (overall/last shell)	6.7 (7.2)
*R*_merge_ (%; overall/last shell)[Link]	12.1 (83.6)
Completeness (%; overall/last shell)	100.0 (100.0)
*I*/*s*(*I*) (overall/last shell)	8.2 (2.0)
Mathews coefficient (Å^3^/Da)	2.80
Solvent content (%)	56.09
Structure refinement	
*R*_factor_ [Link]/*R*_free_ (%)	22.4/25.3
No. of unique reflections (working/test set)	9,979 (973)
Total number of atoms	821
Clashscore	13.99
Average B‐factor (Å^2^)	34.68
Average protein B‐factor (Å^2^)	34.65
Average water B‐factor (Å^2^)	39.60
R.m.s. deviations from standard geometry	
Bonds (Å)	0.007
Angles (º)	1.00
Ramachandran plot statistics	
Most favored regions (%)	93.67
Allowed regions (%)	6.33
Outliers (%)	0.00

*R*_merge_ = Σ|*I* − (*I*)|/Σ(*I*), where *I* is the observed intensity and (*I*) is the average intensity of multiple observations of symmetry‐related positions.

*R*_factor_ = Σ||/*F*_o_|−|*F*_c_||/Σ|*F*_o_|, where |*F*_o_| and |*F*_c_| are observed and calculated structure factor amplitudes, respectively.

A sequence alignment was performed, and a phylogenetic tree was constructed to assess sequence similarities and conservation of residues with other LMW‐PTP with solved structures. *Synechocystis*’ Wzb was shown to be more closely related to the LMW‐PTP of the unicellular eukaryote *E. histolytica* than of the LMW‐PTPs of other bacteria (Supporting Information Figure S11). Likewise, a three dimensional protein structure alignment revealed that the structure of Wzb is highly identical to that of the other LMW‐PTPs, being more closely related to that of *E. histolytica* (PDB entry 3ido) (Linford et al., 2014), followed by *S. aureus* (Gram‐positive; PDB entry 3rof) (Vega et al., 2011) and *E. coli* (Gram‐negative; PDB entry 2wja) (Hagelueken, Huang, Mainprize, Whitfield, & Naismith, 2009), with root mean square deviation (RMSD) values (Å) of 1.04, 1.37, and 1.54, respectively, and an average of 95 superimposable residues.

### 3.3 Wzc is a substrate of the Wzb phosphatase

**Figure 4:**
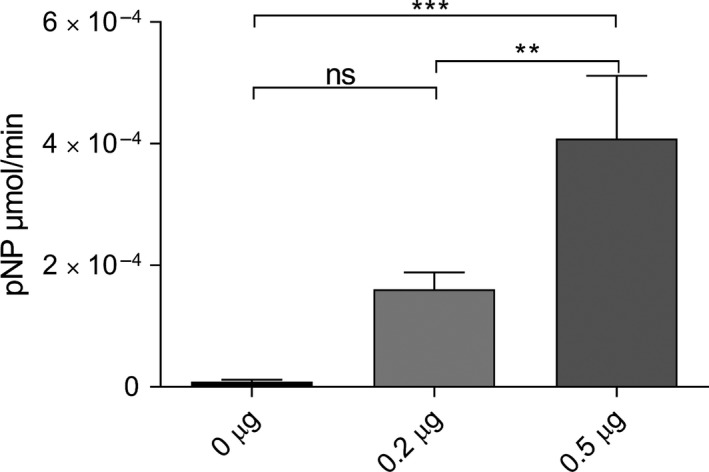
His6‐Wzb phosphatase activity, expressed in µmoles per minute of p‐nitrophenol (pNP), using two different amounts of protein and 10 mM of p‐nitrophenyl phosphate (pNPP) substrate at pH 6.5. Experiments were performed in triplicate. Data are mean ± *SD*. Statistical analysis was performed using one‐way analysis of variance (ANOVA), followed by Tukey's multiple comparisons. Statistically significant differences are identified: **(*p* ≤ 0.01) and ***(*p* ≤ 0.001). Ns: no significant differences

**Figure 5:**
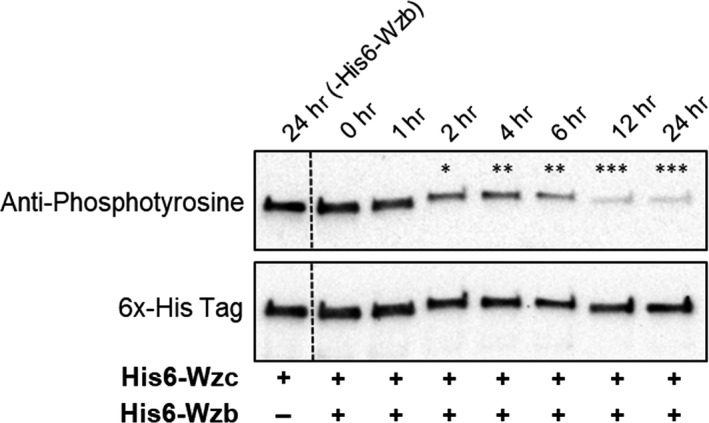
*In vitro* dephosphorylation of His6‐Wzc by His6‐Wzb for 0–24 hr. Samples were analyzed by Western blot using an anti‐phosphotyrosine antibody. Subsequently, membranes were stripped and reprobed with a 6x‐His epitope tag antibody (loading control). Lanes 24 hr (‐His6‐Wzb) refer to samples in which His6‐Wzc was incubated in the absence of His6‐Wzb (control). Experiments were performed in triplicate. Statistical analysis was performed using one‐way analysis of variance (ANOVA), followed by Tukey's multiple comparisons. Significant intensity differences between 0 hr and other time points are identified: *(*p* ≤ 0.05), **(*p* ≤ 0.01), ***(*p* < 0.001). Ns: no significant differences

Our phenotypic characterization of the *Δwzb* and *wzc_Trunc_* mutants raised the possibility that Wzb does not dephosphorylate Wzc. To solve this question, we first used a p‐nitrophenyl phosphate hydrolysis assay to confirm that Wzb functions as a phosphatase (Figure 4), in agreement with our structural analysis (Figure 3) and previous data (Mukhopadhyay & Kennelly, 2011). We then asked whether *Synechocystis*’ Wzc is a substrate for Wzb. Importantly, mass spectrometry analysis of purified Wzc expressed in *E. coli* and solubilized with detergent revealed the presence of phosphorylated tyrosine residues. In particular, the C‐terminal Y‐rich peptide—_740_YYNNRYYDR_748_—shows the presence of a phosphorylation in Y745 and a double phosphorylation at either the Y741 + Y746 pair or at Y745 + Y746 (Supporting Information Figure S12). We used this characteristic of Wzc and incubated the protein with Wzb, assessing the membrane protein phosphorylation load at different time points. In the presence of Wzb, a time‐dependent dephosphorylation of Wzc was observed, with the signal of the anti‐phophotyrosine antibody decreasing significantly after 2‐hr incubation (Figure 5). By MS/MS analysis, we determined that Y745 is one of the residues that undergoes dephosphorylation, with the signal decreasing significantly after incubation of Wzb. In contrast, no Wzc dephosphorylation occurred in the absence of Wzb (Figure 5).These results clearly establish that Wzb is able to interact *in vitro* with the C‐terminal Y‐rich tail of Wzc, suggesting that in the cell, the phosphorylation state of Wzc is dependent on the activity of Wzb.

### 3.4 Wzc has ATPase and autokinase activity

**Figure 6:**
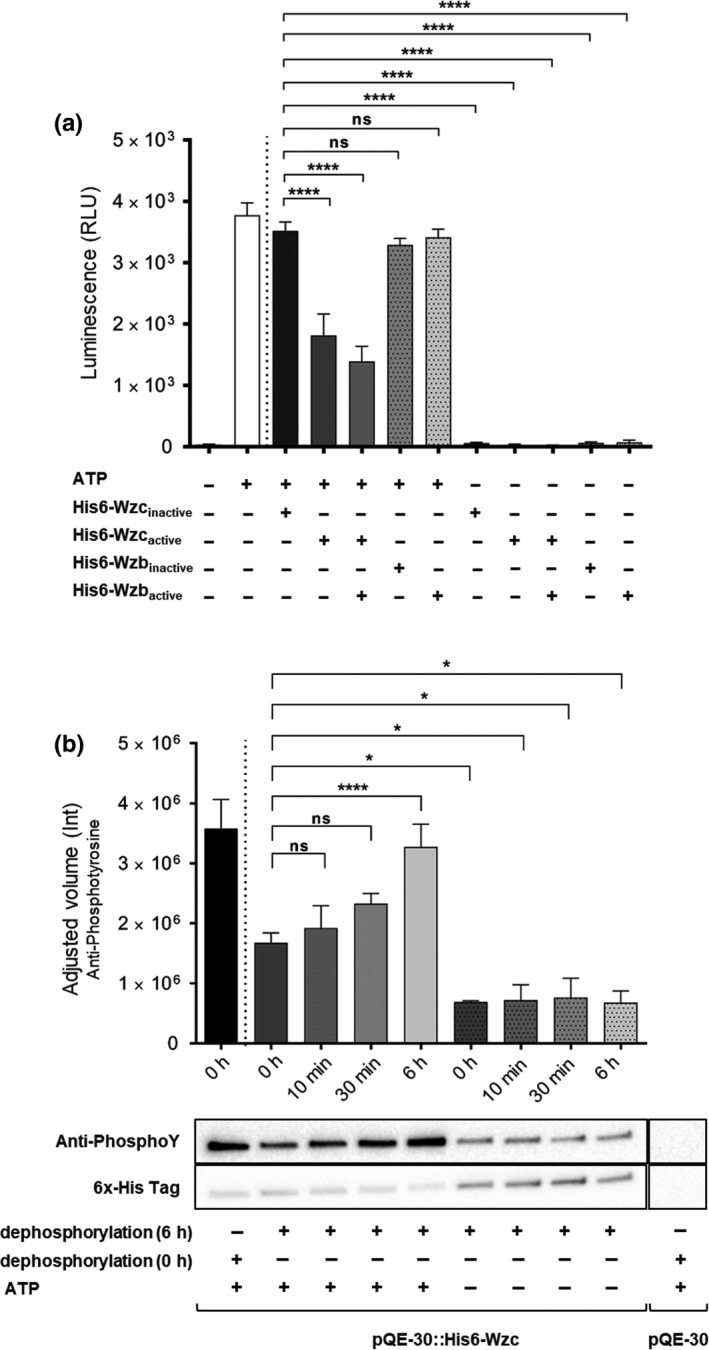
*In vitro* evaluation of His6‐Wzc ATPase (a) and autokinase (b) activities. (a) The ATPase activity was measured using the luciferase assay, being the intensity of the emitted light (Relative Luminescence Unit—RLU) proportional to the ATP concentration in the samples. The presence/absence of ATP, active His6‐Wzc, His6‐Wzc inactivated by heat, His6‐Wzb and/or Wzb inactivated by heat is indicated below. (b) To evaluate the autokinase activity, everted membrane vesicles of *Escherichia coli* cells containing pQE‐30::His6‐Wzc or pQE‐30 were isolated, dephosphorylated for 0 or 6 hr with His6‐Wzb, and dialyzed to remove the dephosphorylation buffer and most of the phosphatase, before incubation with or without 200 μM ATP for 0 hr, 10 min, 30 min, or 6 hr. The time of dephosphorylation and the presence/absence ATP is indicated below. Samples were analyzed by Western blot using an anti‐phosphotyrosine antibody. Subsequently, membranes were stripped and reprobed with a 6x‐His epitope tag antibody (loading control). The ratio between the two signals (adjusted volume) is shown. Experiments were performed in triplicate. Data are means ± *SD*. Statistical analysis was performed using one‐way analysis of variance (ANOVA), followed by Tukey's multiple comparisons. Statistically significant differences between inactivated Wzc in the presence of ATP (a) or dephosphorylation for 6 hr followed by incubation with ATP for 0 hr (b) and other conditions are identified: *(*p* ≤ 0.05), **(*p* ≤ 0.01), ***(*p* ≤ 0.001), ****(*p* ≤ 0.0001). Ns: no significant differences

It was previously suggested that Wzc is a BY‐kinase (Mijakovic et al., 2016; Pereira et al., 2013, 2015). To demonstrate that this protein functions as a kinase, we investigated its ability to hydrolyze ATP. ATPase activity was evaluated using a bioluminescence assay in which the signal intensity is proportional to the ATP concentration. A significant reduction of the signal intensity was observed for the reactions containing either Wzc or both Wzc and Wzb, compared to those in which Wzc was previously heat inactivated (Figure 6a). These results provide evidence that Wzc is capable of ATP binding and hydrolysis and that the presence of Wzb does not significantly impair this activity. To determine whether Wzc also displays autokinase activity, we tested its ability to autophosphorylate in the presence of ATP. For this, we used everted membrane vesicles (EMVs) of *E. coli* overexpressing *Synechocystis*’ Wzc, which were pre‐incubated with Wzb to generate dephosphorylated Wzc. To take into account possible differences in the amount of protein loaded, the signal obtained for the anti‐phosphotyrosine antibody was normalized by that obtained for the 6x‐His epitope tag antibody. The results revealed an ATP‐dependent increase in the level of phosphorylated Y residues of Wzc over time (Figure 6b). To confirm that the immunoblot signal was due to the *Synechocystis*’ Wzc and not the native *E. coli* Wzc, EMVs isolated from cells transformed with the empty pQE‐30 vector were also included in the assay. Altogether, the ATPase activity and the increase in phosphorylation strongly suggest autokinase activity.

## 4 DISCUSSION

The results obtained in this work clearly show that *Synechocystis*’ Wzc and Wzb are involved in the production of EPS. Importantly, the amount of total carbohydrates did not vary in ∆*wzc* and ∆*wzb*, consistent with an intracellular accumulation of the polysaccharides and supporting the idea that these proteins are mainly involved in the later steps of polymer assembly and export. Neither Wzc nor Wzb are essential for EPS production, as the polymer(s) is still produced in their absence. This is consistent with the hypothesis of functional redundancy, either owing to the existence of multiple copies for some of the EPS‐related genes/proteins and/or a crosstalk between the components of the different assembly and export pathways (Pereira et al., 2015). This hypothesis also helps to explain the lack of phenotypes for ∆*wzy* (∆*sll0737*), ∆*wzx* (∆*sll5049*), ∆*kpsM* (∆*slr2107*), and ∆*kpsM*∆*wzy* (∆*slr2107*∆*sll0737*).

We have established that the Wzb phosphatase has a classical LMW‐PTPs conformation and that Wzc has the functional properties of a BY‐kinase, showing ATPase and autophosphorylation activity. We also provide evidence that Wzc is a substrate of Wzb and that the Wzb‐mediated dephosphorylation occurs at the C‐terminal Y‐rich loop of Wzc. These findings fit well with the previously proposed roles for the two proteins and strongly indicate that tyrosine phosphorylation of Wzc and its dephosphorylation by Wzb is a mechanism of regulation of EPS production. Two features of the Wzb structure are particularly relevant for its functional properties: (a) it possesses the second Y of the DPYY motif (Y127) that is absent in most prokaryotic LMW‐PTPs (Bohmer, Szedlacsek, Tabernero, Ostman, & Hertog, 2013; Lescop et al., 2006; Standish & Morona, 2014). While the first tyrosine usually interacts with the aromatic ring of the substrate, being crucial for the interaction between LMW‐PTPs and the BY‐kinases (Lescop et al., 2006; Standish & Morona, 2014), the second tyrosine seems to play a role in substrate specificity (Bucciantini et al., 1999; Raugei, Ramponi, & Chiarugi, 2002). (b) *Synechocystis*’ Wzb possesses an aromatic residue—histidine (H45)—in the catalytic site instead of the hydrophobic leucine that occupies this position in most prokaryotic LMW‐PTPs (Lescop et al., 2006; Linford et al., 2014). Based on these characteristics, *Synechocystis* Wzb can be classified as a class I or eukaryotic‐like LMW‐PTP and is therefore likely to present a substrate recognition mechanism different from that of *E. coli* Wzb, a class II or prokaryotic‐like LMW‐PTPs (Lescop et al., 2006). These differences may contribute to the higher substrate affinity of the *Synechocystis*’ Wzb compared to the *E. coli* homolog using pNPP as substrate (Lescop et al., 2006; Mukhopadhyay & Kennelly, 2011) and to the ability of *Synechocystis*’ Wzb to dephosphorylate other substrates (Mukhopadhyay & Kennelly, 2011). In addition, since most of the phosphatases involved in the biosynthesis of capsules and/or EPS are class II LMW‐PTPs, it is likely that the structural features of *Synechocystis*’ Wzb reflect differences in EPS production.

Strikingly, although our results indicate that Wzb regulates the function of Wzc through dephosphorylation, the two proteins appear to have different roles in EPS production. Wzc plays a role in both CPS and RPS production, as shown here and previously (Jittawuttipoka et al., 2013), while Wzb participates in RPS production only. Our *wzc*_Trunc_ mutant, which lacks the protein region where Wzb acts, provides some insights into these differences. The phenotype of *wzc*_Trunc_, together with the results obtained for ∆*wzc* and ∆*wzb*, supports previous indications that the production of CPS and RPS is, at least, divergent processes (Micheletti et al., 2008), relying on different proteins and/or different contributions of the same proteins (Jittawuttipoka et al., 2013). For example, it is possible that the assembly and export of CPS and RPS are regulated by different signals involving the Y‐rich region of Wzc. This is true for *E. coli* K30 capsule (CPS‐like) and K‐12 colanic acid (RPS‐like), with the production of the capsule depending on Wzc undergoing cycles of phosphorylation/dephosphorylation while the production of colanic acid being negatively regulated by phosphorylated Wzc (Paiment, Hocking, & Whitfield, 2002). However, it is important to keep in mind that the C‐terminal Y‐rich region is not universally present in the Wzc proteins of EPS‐producing bacteria, including *Xanthomonas campestris*’ GumC (Cuthbertson et al., 2009) and the Wzc homologs of the highly efficient EPS producer *Cyanothece* sp. CCY 0110 (Pereira et al., 2013).

In addition, our results show that Wzc and Wzb influence EPS composition. Three factors are likely to contribute to the monosaccharidic differences observed for the RPS of the mutants versus wild type: (a) *Synechocystis*’ Wzc and Wzb interact with other proteins that may directly or indirectly affect monosaccharide and/or sugar nucleotide metabolism (Mukhopadhyay & Kennelly, 2011; Sato et al., 2007). In particular, Wzb substrates include proteins involved in the photosynthetic process (Mukhopadhyay & Kennelly, 2011) and changes in Wzb activity will likely affect central carbon metabolism. It is therefore not surprising that the RPS of ∆*wzb* displays the highest number of differences relative to wild type composition; (b) mutations may lead to differences in the affinity of the periplasm–spanning complex for monosaccharides. This explanation has been suggested for a mutant in the ATP‐binding component (KpsT; Sll0982) of an EPS‐related ABC transporter that also produces EPS enriched in rhamnose (Fisher et al., 2013). (c) The increase in the rhamnose content may be related to the genomic context of *wzc*, since this gene is present in the vicinity of *slr0985* that encodes one of the enzymes involved in the dTDP‐l‐rhamnose biosynthesis, the dTDP‐4‐dehydrorhamnose 3,5‐epimerase. Interestingly, the same is observed for *kpsT* (*sll0982*) and its cognate permease component *KpsM* (*sll0977*). In bacteria, l‐rhamnose is a rare sugar that is most frequently found in the EPS and O‐antigen of LPS and this genetic organization is not uncommon (Boels et al., 2004) (Giraud & Naismith, 2000; Roca et al., 2015). Further studies are necessary to discern the mechanistic role of Wzb and Wzc on the composition of EPS.

Overall, the results obtained in this study emphasize the complexity of EPS production in cyanobacteria, supporting previous hypothesis of functional redundancy and crosstalk between different pathways, and provide novel data on the specific function of two proteins—Wzc and Wzb—in *Synechocystis*. These findings provide a robust basis for future studies aiming at further elucidate cyanobacterial EPS production with the ultimate goal of establishing these organisms as sustainable platforms for the production of large amounts of these polymers and/or polymers with the desired characteristics for the industrial toolbox.

## CONFLICT OF INTEREST

The authors declare that they have no conflict of interest.

## AUTHORS CONTRIBUTION

SBP, MS, JMC, LG, and PT designed the research; SBP, MS, JPL, CF, ZB, CE, and FR performed research; SBP, MS, JPL, RM, RDP, LG, JMC, and PT analyzed and interpreted data; SBP, MS, JMC, and PT wrote the manuscript.

## ETHICS STATEMENT

None required.

## Data Availability

The atomic coordinates of Wzb have been deposited in the Protein Data Bank, www.pdb.org (PDB ID 5O7B). All data supporting this study are provided as Supporting Information Appendix S1 accompanying this paper.
