# Supplementary material for: The role of the tyrosine kinase Wzc (Sll0923) and the phosphatase Wzb (Slr0328) in the production of extracellular polymeric substances (EPS) by Synechocystis PCC 6803
Source: Microbiologyopen. 2019 Jan 23;8(6):e00753. doi: 10.1002/mbo3.753 (PMC6562117; doi:10.1002/mbo3.753)
Supplement: Supplementary file 1 [file MBO3-8-e00753-s001.docx]

**Supporting information**

**The tyrosine kinase Wzc (Sll0923) and the phosphatase Wzb (Slr0328) play a role in the production of extracellular polymeric substances (EPS) in *Synechocystis* PCC 6803**

Sara B. Pereira,^1,2†^ Marina Santos,^1,2,3†^ José P. Leite,^1,2,3^ Carlos Flores,^1,2,3^ Carina Eisfeld,^1,2‡^ Zsófia Büttel,^2§^ Rita Mota,^1,2,3^ Federico Rossi,^4^ Roberto De Philippis,^4^ Luís Gales,^1,2,3^ João H. Morais-Cabral,^1,2^ and Paula Tamagnini^1,2,5*^

^1^i3S - Instituto de Investigação e Inovação em Saúde, Universidade do Porto, Porto Portugal.

^2^IBMC - Instituto de Biologia Molecular e Celular, Universidade do Porto, Porto, Portugal.

^3^ICBAS – Instituto de Ciências Biomédicas Abel Salazar, Porto, Portugal.

^4^Department of Agrifood Production and Environmental Sciences, University of Florence, Florence, Italy.

^5^Faculdade de Ciências, Departamento de Biologia, Universidade do Porto, Porto, Portugal.

^*^For correspondence. E-mail pmtamagn@ibmc.up.pt; Tel. +351 220 408 800.

^†^These authors contributed equally to this work.

^‡^Present address: Department of Water Management, Delft University of Technology, Delft, The Netherlands.

^§^Present address: Molecular Microbiology, Groningen Biomolecular Sciences and Biotechnology Institute, University of Groningen, Groningen, The Netherlands.

**Appendix S1 : PCR protocols**

*Construction of pGDsll0727, pGDsll5049, pGDslr2107, pGDslr0328* and *pGDsll0923*_Trunc_

For the amplification of *sll0727*, *sll5049*, *slr2107* and *slr0328* flanking regions, each PCR reaction mixture (50 µL) contained 1.5 u of *Pfu* DNA polymerase (Thermo Scientific), 1x reaction buffer, 250 µM of each deoxyribonucleotide triphosphate, 200 nM of each primer (primer pairs 5O/5I or 3O/3I), and 7 ng of *Synechocystis* genomic DNA. For the amplification of *sll0923*_Trunc_ flanking regions, each PCR reaction mixture (50 µL) contained 1x Accuzyme reaction mix (Bioline), 200 nM of each primer (primer pairs 5O/5I or 3O/3I), and 12.6 ng of *Synechocystis* genomic DNA. The PCR profile was: 1 min at 94°C followed by 35 cycles of 30 s at 94°C, 45 s at 52°C (*sll0737*) or 48ºC (*sll5049* and *slr2107*) and 90 s at 72°C, and a final extension at 72°C for 7 min. For *slr0328*, the PCR profile was: 3 min at 94°C followed by 40 cycles of 1 min at 94°C, 1min at 48°C and 1min at 72°C, and a final extension at 72°C for 7 min. For *pGDsll0923*_Trunc_ the PCR profile was: 1 min at 95°C followed by 35 cycles of 15 s at 95°C, 15 s at 52°C and 2 min at 72°C, and a final extension at 72°C for 7 min.

For the Overlap PCR, each reaction mixture (50 µL) contained 1.25 u of GoTaq^®^ Flexi DNA polymerase (Promega), 1x Green GoTaq^®^ Flexi Buffer, 1.5 µM MgSO_4,_ 250 µM of each deoxyribonucleotide triphosphate, 125 nM of each outer primer (5O and 3O), and 80 ng of each purified DNA fragment. The PCR profile used for *sll0737* and *sll5049* was: 5 min at 95°C, 10 cycles of 30 s at 95°C, 45 s at 48°C and 90 s at 72°C, followed by 30 cycles of 30 s 95°C 45 s at 55°C and 90 s at 72°C and a final extension at 72°C for 7 min. The PCR profile for *slr2107* was: 5 min at 95°C followed by 40 cycles of 30 s at 95 °C, 45 s at 55 °C and 90 s at 72 °C, and a final extension at 72 °C for 7 min. The PCR profile for *slr0328* was: 5 min at 94°C, followed by 40 cycles of 1 min at 94°C, 110 s at 56°C and 110 s at 72°C and a final extension at 72 °C for 7 min.

The PCR profile for *sll0923*_Trunc_ was: 5 min at 95°C, followed by 10 cycles of 30 s at 95°C, 45 s at 48°C and 2 min at 72°C, 30 cycles of 30 s at 95°C, 45 s at 56°C and 2 min at 72°C and a final extension at 72 °C for 7 min.

*Amplification Km resistance cassette from pKm.1*

The PCR reaction mixture (50 µL) contained 1.25 u of *Pfu* polymerase (Thermo Scientific), 1x reaction buffer, 250 µM of each deoxyribonucleotide triphosphate, 200 nM of each primer and 10 ng of template DNA. The PCR profile was: 1 min at 95°C followed by 35 cycles of 60 s at 94°C, 45 s at 52°C and 3 min at 72°C, and a final extension at 72°C for 7 min.

*Construction of pS351sll0923*

For the amplification of a fragment covering *wzc* and its native promoter (P*_wzc_*) and RBS, each PCR reaction mixture (20 µL) contained 0.4 u of *Phusion* DNA polymerase (Thermo Scientific), 1x *Phusion* HF reaction buffer, 200 µM of each deoxyribonucleotide triphosphate, 500 nM of each primer and 15 ng of *Synechocystis* genomic DNA. The PCR profile was: 30 s at 98°C followed by 35 cycles of 20 s at 98°C, 40 s at 68°C and 50 s at 72°C, and a final extension at 72°C for 10 s.

*Construction of* pS351sll0923_Trunc_

For the amplification of a fragment covering *wzc*_Trunc_ and its native promoter (P*_wzc_*) and RBS, each PCR reaction mixture (50 µL) contained 1 u of *Phusion* DNA polymerase (Thermo Scientific), 1x *Phusion* HF reaction buffer, 200 µM of each deoxyribonucleotide triphosphate, 250 nM of each primer and 15 ng of *Synechocystis* genomic DNA. The PCR profile was: 30 s at 98°C followed by 35 cycles of 10 s at 98°C, 30 s at 62°C and 90 s at 72°C, and a final extension at 72°C for 10 s.

*Construction of pS351slr0328*

For the amplification of a fragment covering *wzb* and incorporating the synthetic RBS BBa_B0030, each PCR reaction mixture (20 µL) contained 0.4 u of *Phusion* DNA polymerase (Thermo Scientific), 1x *Phusion* HF reaction buffer, 200 µM of each deoxyribonucleotide triphosphate, 500 nM of each primer and 15 ng of *Synechocystis* genomic DNA. The PCR profile was: 30 s at 98°C followed by 35 cycles of 20 s at 98°C, 40 s at 68°C and 15 s at 72°C, and a final extension at 72°C for 10 s.

**Table S1.** List of organisms and plasmids used/generated in this work.

| **Organism name/Genotype** | **Description** | **Source** |
| --- | --- | --- |
| *Escherichia coli* DH5α | Transformation/cloning strain. | Invitrogen |
| *Escherichia coli* XL1-Blue | Transformation/cloning strain. | Agilent |
| *Escherichia coli* M15 (pREP4) | Protein overexpression/purification strain. | QIAGEN |
| *Synechocystis* sp. PCC 6803 | Wild type strain. | Pasteur Culture Collection |
| ∆*wzy* | *Synechocystis* mutant with *sll0727* replaced by a Km resistance cassette. | This work |
| ∆*wzx* | *Synechocystis* mutant with *sll5049* replaced by a Km resistance cassette. | This work |
| ∆*kpsM* | *Synechocystis* mutant with *slr2107* replaced by a Km resistance cassette. | This work |
| ∆*kpsM*∆*wzy* | *Synechocystis* mutant with *slr2107* and *sll0727* replaced by a Km or a Sm/Sp cassettes, respectively. | This work |
| ∆*wzc* | *Synechocystis* mutant with *sll0923* replaced by a Km resistance cassette. | This work |
| ∆*wzb* | *Synechocystis* mutant with *slr0328* replaced by a Km resistance cassette. | This work |
| ∆*wzc*∆*wzb* | *Synechocystis* mutant with *sll0923* and *slr0328* replaced by a Km or a Sm/Sp cassettes, respectively. | This work |
| ∆*wzb* pS351slr0328 | *Synechocystis ∆wzb* mutant complemented with the replicative plasmid pS351sll0923. | This work |
| ∆*wzc* pS351sll0923 | *Synechocystis ∆wzc* mutant complemented with the replicative plasmid pS351sll0923. | This work |
| *Wzc*_trunc_ | *Synechocystis* mutant with *sll0923* replaced by a truncated form of the gene (from 1 to 2196 bp) and a Km resistance cassette. | This work |
| Δ*wzc* pS351sll0923_Trunc_ | *Synechocystis ∆wzc* mutant complemented with the replicative plasmid pS351sll0923_Trun_. | This work |
| **Plasmid** | **Description** | **Source** |
| pGEM^®^-T easy | T/A cloning vector. | Promega |
| pSEVA351 | Replicative shuttle vector for *Synechocystis* transformation. | SEVA-DB (Silva-Rocha *et al.*, 2013) |
| pSEVA481 | Source of the Sm resistance cassette. | SEVA-DB (Silva-Rocha *et al.*, 2013) |
| pKm.1 | pGEM-T easy with the Km resistance cassette. | (Pinto *et al.*, 2015) |
| pGDsll0727 | pGEM-T easy with *sll0727* and its flanking sequences, where the *sll0727* coding sequence (from 14 to 2475 bp) was replaced by a *Xma*I site. | This work |
| pGDsll0727.Km | pGDsll0727 with a Km resistance cassette inserted into the *Xma*I site. | This work |
| pGDsll0727.Sm | pGDsll0727 with a Sm/Sp resistance cassette inserted into the *Sma*I site. | This work |
| pGDsll0549 | pGEM-T easy with *sll5049* and its flanking sequences, where the *sll5049* coding sequence (from 145 to 1310 bp) was replaced by a *Xma*I site. | This work |
| pGDsll0549.Km | pGDsll0549 with a Km resistance cassette inserted into the *Xma*I site. | This work |
| pGDslr2107 | pGEM-T easy with *slr2107* and its flanking sequences, where the *slr2107* coding sequence (from 52 to 821 bp) was replaced by a *Xma*I site. | This work |
| pGDslr2107.Km | pGDslr2107 with a Km resistance cassette inserted into the *Xma*I site. | This work |
| pGDslr0328 | pGEM-T easy with *slr0328* and its flanking sequences, where the *slr0328* coding sequence (from 214 to 383 bp) was replaced by a *Xma*I site. | This work |
| pGDslr0328.Km | pGDslr0328 with a Km resistance cassette inserted into the *Xma*I site. | This work |
| pGDslr0328.Sm | pGDslr0328 with a Sm/Sp resistance cassette inserted into the *Xma*I site. | This work |
| pDsll0923::Km^r^ | pDsll0923 with a Km resistance cassette inserted into a *SmaI* site. | (Jittawuttipoka *et al.*, 2013) |
| pGDsll0923_Trunc_ | pGEM-T easy with *sll0923* (from 1461 to 2193 bp) and its flanking sequence, where the last 78 bp of *sll0923* were replaced by a *Xma*I site. | This work |
| pGDsll0923_Trunc_.Km | pGDsll0923_Trunc_ with a Km resistance cassette inserted into the *Xma*I site. | This work |
| pSB1C3 | Source of the promoter of *rnpB* (P*_rnpB_*). | Registry of Standard Biological Parts (http://parts.igem.org). |
| pS351P*_rnpB_* | pSEVA351 with P*_rnpB_*. | This work |
| pS351slr0328 | pSEVA351 with slr0328 downstream the synthetic RBS BBa_B0030, under the control of P*_rnpB_*. | This work |
| pS351sll0923 | pSEVA351 with *sll0923* downstream its native RBS and under the control of its native promoter P*_wzc_* (-230 to +123). | This work |
| pS351sll0923_Trunc_ | pSEVA351 with a truncated *sll0923* (from 1 to 2196 bp) downstream its native RBS and under the control of P*_wzc_*. | This work |
| pQE-30::His6-Wzc | pQE-30 with *sll0923* with 6xHis tag coding sequence at 5’. | This work |
| pQE-30::His6-Wzc_trunc_ | pQE-30 with a truncated *sll0923* (from 1 to 2196 bp) with 6xHis tag coding sequence at 5’. | This work |

**Table S2.** Oligonucleotides used in this work.

| **Name** | **Sequence (5’-3’)** | **Purpose** | **Reference** |
| --- | --- | --- | --- |
| sll0737.5O | TGTTGAGGTGGAAGCAGCGGAGCCCAAAGG | Amplification of flanking region;  5I and 3I: overlap PCR | This work |
| sll0737.5I | GAACCAAGTTACCAGCCCGGGAATCGGCGGCCATACTGGGCAATACTCACAGG |  | This work |
| sll0737.3I | TATGGCCGCCGATTCCCGGGCTGGTAACTTGGTTCCCGTTTATGTTGCCTTCCC |  | This work |
| sll0737.3O | CTTCCTCTGCATACTGCCCAGCGGGAACAC |  | This work |
| sll5049.5O | TTGCCGAGTTTCGCCGAAGGTTTACCG |  | This work |
| sll5049.5I | ATCGGTAAACCCAGTCCCGGGTACCAACGCCATCAGGCCAAACATTTCCG |  | This work |
| sll5049.3I | CCTGATGGCGTTGGTACCCGGGACTGGGTTTACCGATTGAAGCGTTATGG |  | This work |
| sll5049.3O | TCAACACTATTGGGCACAAGGGAGACTTGGG |  | This work |
| slr2107.5O | CGCAGGCAATTGAAGATATAAAGTGGTGGATTCAAC |  | This work |
| slr2107.5I | ACGGCATCGCCAAACCCGGGACGACTCTCCGGCGTATAAACAATGACTGGTTGCG |  | This work |
| slr2107.3I | TACGCCGGAGAGTCGTCCCGGGTTTGGCGATGCCGTTTATTGTTGAG |  | This work |
| slr2107.3O | CAATCTCCGCAAACGCCACCACATCCTCAAATCG |  | This work |
| slr0328.5O | CACTTCCTTTGCCGTCAAAGTTGCTTCCAT |  | This work |
| slr0328.5I | CTCAAAACCAGCCTGCCCGGGCTGTCTAGCTCTTCCCTGCACCCGATAA |  | This work |
| slr0328.3I | GGGAAGAGCTAGACAGCCCGGGCAGGCTGGTTTTGAGCATGTGATTGATT |  | This work |
| slr0328.3O | CCGTAGGGTTTGGGCAGAAGCATGTTGCT |  | This work |
| sll0923.5O | GAGAGCTAGTCAGCACCACACCATCCT |  | This work |
| sll0923.5I | CTGTGCTCCCGATCCCGGGTTATTAGCCGGACGTAGAAGTGATAGCATTGG |  | This work |
| sll0923.3I | ACGTCCGGCTAATAACCCGGGATCGGGAGCACAGAACCCCACCTTGCGG |  | This work |
| sll0923.3O | CGGCGAGATTTAGACTTGCCTTGGCTAGA |  | This work |
| Km.KmScFwd | CTGACCCCGGGTGAATGTCAGCTACTGG | Amplification of Km resistance cassette | (Pinto *et al.*, 2015) |
| KmRev | CAAACCCGGGCGATTTACTTTTCGACCTC |  | (Pinto *et al.*, 2015) |
| sll0923_compF1 | TCTAGAGGTTCTGCGTTAGCATCACA | Amplification of *wzc* or *wzc*_Trunc_, native promoter and RBS | This work |
| sll0923_compR1 | GCACTAGTCGATTAGCTCAGTTGGTAGA | Amplification of *wzc*, native promoter and RBS | This work |
| sll0923.RTrunc | GCACTAGTCTAGCCGGACGTAGAAGTGATA | Amplification of *wzc*_Trunc_, native promoter and RBS | This work |
| slr0328_compF | gtttcttcgaattcgcggccgcttctagagattaaagaggagaaaactagatgaaattgttatttgtttg | Amplification of *wzb* introducing B0030 | This work |
| slr0328_compR | gtttcttcctgcagcggccgctactagtactaattaaccaattccttgc | Amplification of *wzb* | This work |
| sll0738F | GCTTGGTTTTGGCTACTGATCCT | Amplification of Southern blot probes | This work |
| sll0738R | GGCGTAAACTATCCCAGCATC |  | This work |
| sll5048F | CAGCCTCTACCTAAACCTGGA |  | This work |
| sll5048R | GAATCGCCAAATCCGTTCCT |  | This work |
| slr2108F | CGGTAGTGATGTGGTGCTGT |  | This work |
| slr2108R | GCCGTTTCACAAATGCGAGT |  | This work |
| sll0923F_SB | CCGCTAAATCCAAAGGCGA |  | This work |
| sll0923R_SB | CCCAGGCAGACAATTAGGAT |  | This work |
| sll0923F2_SB | CTGAAGCCAGTAGCAGCCAAC |  | This work |
| sll0923R2_SB | CGCTCTACCAACTGAGCTAATC |  | This work |
| slr0328F_SB | ccattgccatcctggtcta |  | This work |
| slr0328R_SB | ccacatggtagctggaagta |  | This work |
| Ovslr0328F | ttttgGCATGCaaattgttatttgtttgtttagg | *wzb* amplification | This work |
| Ovslr0328R | caacCTGCAGctaattaaccaattccttgccc |  | This work |
| OvSll0923F | caagGGATCCacctacagttacccagaattg | *wzc* amplification | This work |
| OvSll0923R | ctcCTGCAGctaattgtctgcctgggca |  | This work |
| sll0737F(Ra) | CGGTTAGTTCCCCTTCATCCACT | RT-PCR | This work |
| sll0737RO | GGGTAAACCACAGCAAAAGACTAA |  | This work |
| slr1515F | GGCAGTGTGCTCCATCGTTT |  | This work |
| slr1515R | CCGAAATGCACCAGTAGGCAA |  | This work |
| slr0728F | GCAACTCAATACCCTAGCTTGGC |  | This work |
| slr0728R | GGAGGAAGATTGAAGCGGTTAC |  | This work |
| sll5047F | TGTGACCGATAGCCTCTGGA |  | This work |
| sll5047R | CTGATTTCGTGCCTGCCCTA |  | This work |
| slr1074F | TGGCTACATTCTGGCTCTGC |  | This work |
| slr1074R | CCGTCTTGTAAAGGCGATGC |  | This work |
| sll5049F(Ra) | CCATTCGTGGCACCATTTGGACT |  | This work |
| sll5049RI | GCCGTGTTGAGAAAACTAGGCT |  | This work |
| slr1543F | TATGGGTGGCGGGACTAAGA |  | This work |
| slr1543R | CCCAATAGCCAGCCCAAGAT |  | This work |
| slr0896F | TTTGGCACAAACCCCTCCAT |  | This work |
| slr0896R | ACCAATACTCACCAAGGCCG |  | This work |
| slr0488F | AAACCGTTACCACCCTGGTC |  | This work |
| slr0488R | GCGACCCCTAAACCCAGAAT |  | This work |
| slr2107F(Ra) | GACCCATCGTCAATCGCAAC |  | This work |
| slr2107RO | CCGTCACAATCGGTGGCAAA |  | This work |
| slr0977F | CGCACGGAGCGTCAGTATT |  | This work |
| slr0977RO | CCGCAAACACCAGAATGGGAT |  | This work |
| sll0574F | CGAAAGCACAACCAACCCAG |  | This work |
| sll0574R | GCCATTACCCCTATTGGCGA |  | This work |

Underlined base pairs correspond to restriction sites.


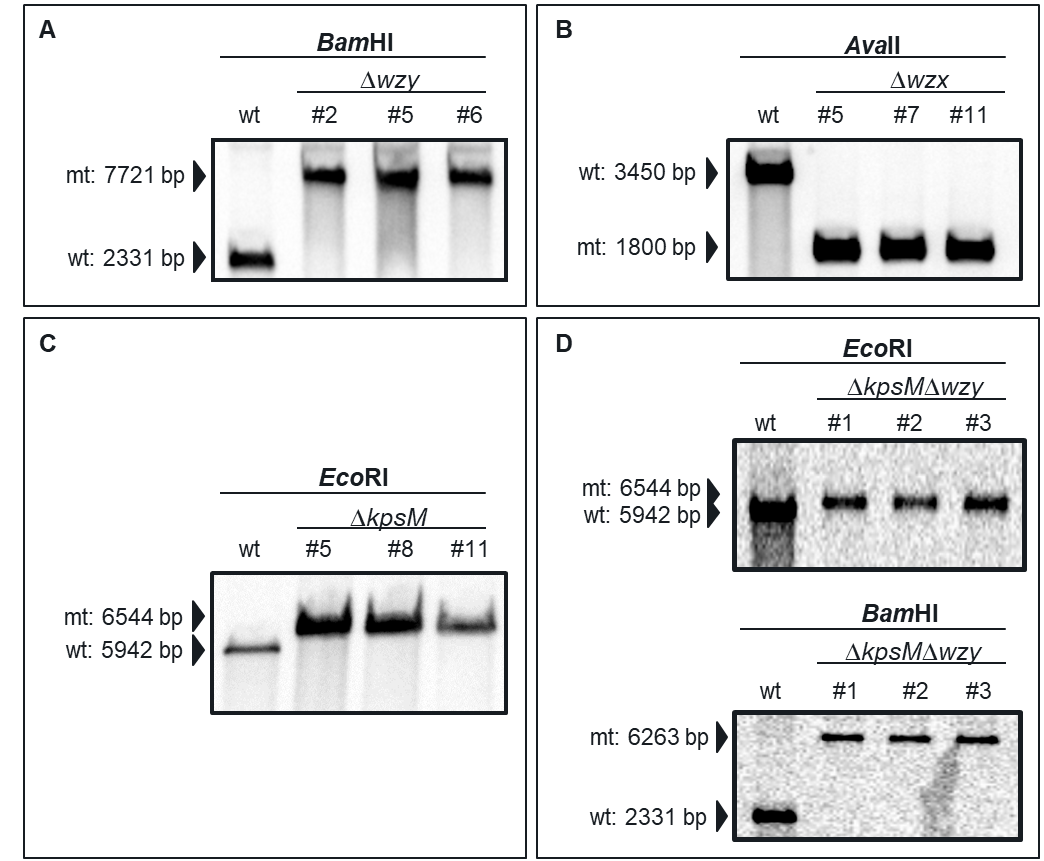


**Fig. S1.** Southern blot analysis confirming the segregation of the *Synechocystis* sp. PCC 6803 mutants (A) ∆*wzy*, (B) ∆*wzx*, (C) ∆*kpsM* and (D) ∆*kpsM*∆*wzy* (∆*kpsM* segregation – upper blot; ∆*wzy* segregation – lower blot). The DNAs were digested with the endonuclease indicated. A dioxigenin labeled probe covering the 3’ flanking region of *wzy*, *wzx* or *kpsM*, repectively was used. The sizes of the DNA fragments are indicated. wt – wild type; # clone tested.

**
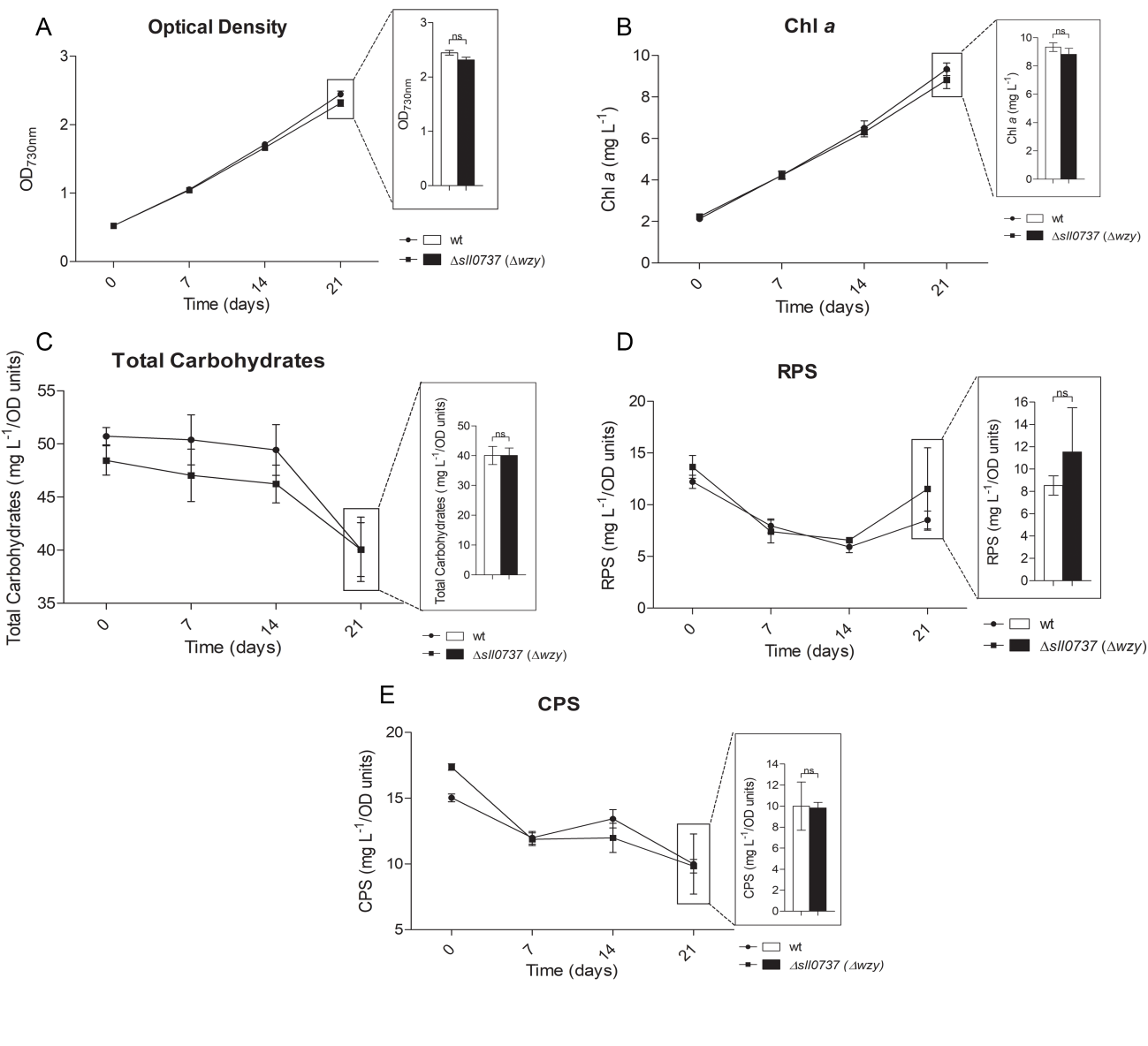
**

**Fig. S2.** Characterization of *Synechocystis* sp. PCC 6803 wild type and Δ*wzy* mutant in terms of growth [(A) optical density at λ=730nm (OD_730nm_) and (B) µg of chlorophyll *a* per mL of culture (Chl *a*)], and production of (C) total carbohydrates, (D) released polysaccharides (RPS) and (E) capsular polysaccharides (CPS) expressed as mg per OD730_nm_ units. Cells were grown in BG11 medium at 30 ºC, under a 12 h light (50 µE m^-2^ s^-1^)/12 h dark regimen at 150 rpm. Experiments were performed in triplicate. Data are means ± SD. Statistical analysis performed using one-way analysis of variance (ANOVA), followed by Tukey's multiple comparisons is presented for the last time point. Ns: no significant differences.

**
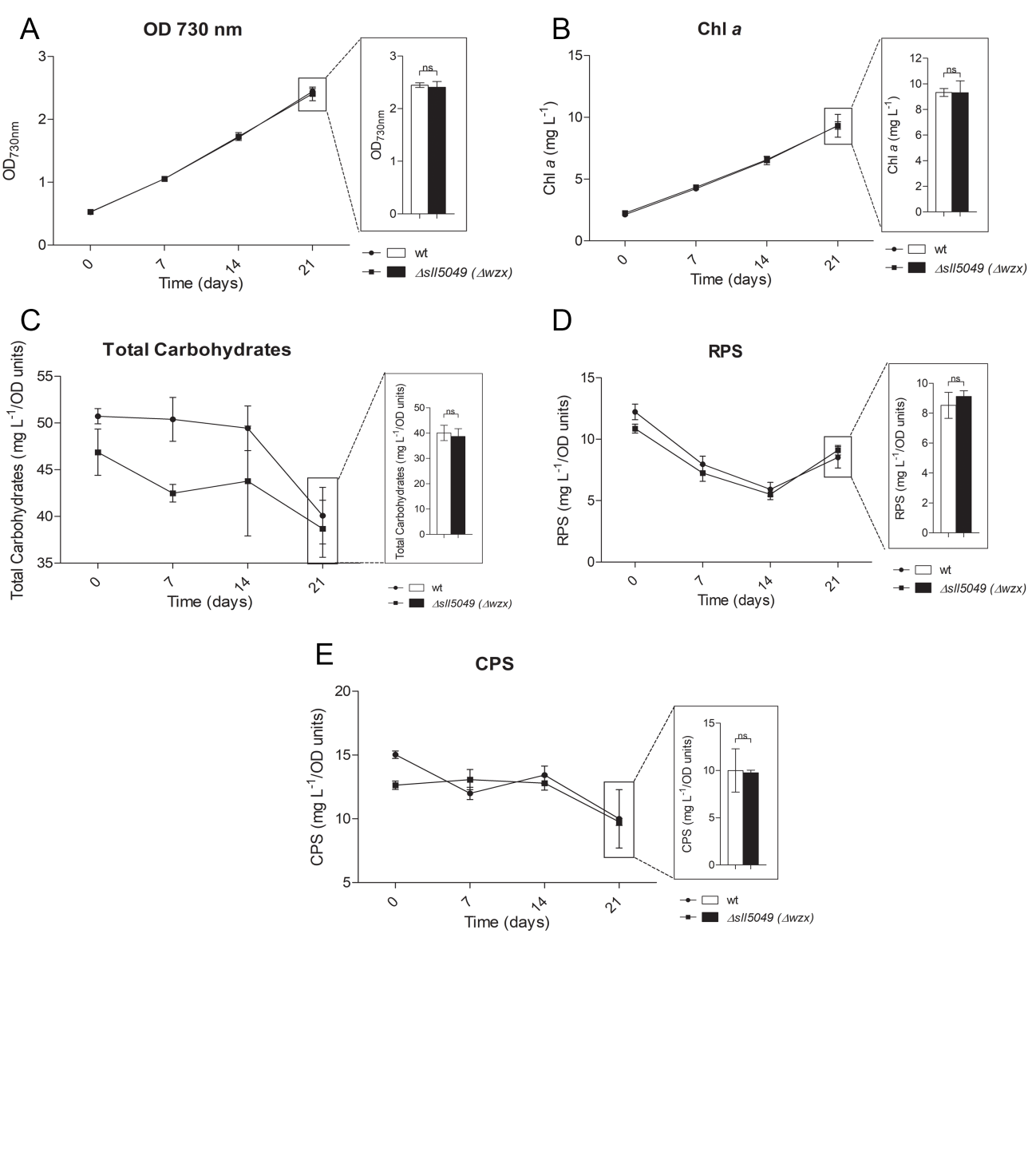
**

**Fig. S3.** Characterization of *Synechocystis* sp. PCC 6803 wild type and Δ*wzx* mutant in terms of growth [ (A) optical density at λ=730nm (OD_730nm_) and (B) µg of chlorophyll *a* per mL of culture (Chl *a*)], and production of (C) total carbohydrates, (D) released polysaccharides (RPS) and (E) capsular polysaccharides (CPS) expressed as mg per OD730_nm_ units. Cells were grown in BG11 medium at 30 ºC, under a 12 h light (50 µE m^-2^ s^-1^)/12 h dark regimen at 150 rpm. Experiments were performed in triplicate. Data are means ± SD. Statistical analysis performed using one-way analysis of variance (ANOVA), followed by Tukey's multiple comparisons is presented for the last time point. Ns: no significant differences.

**
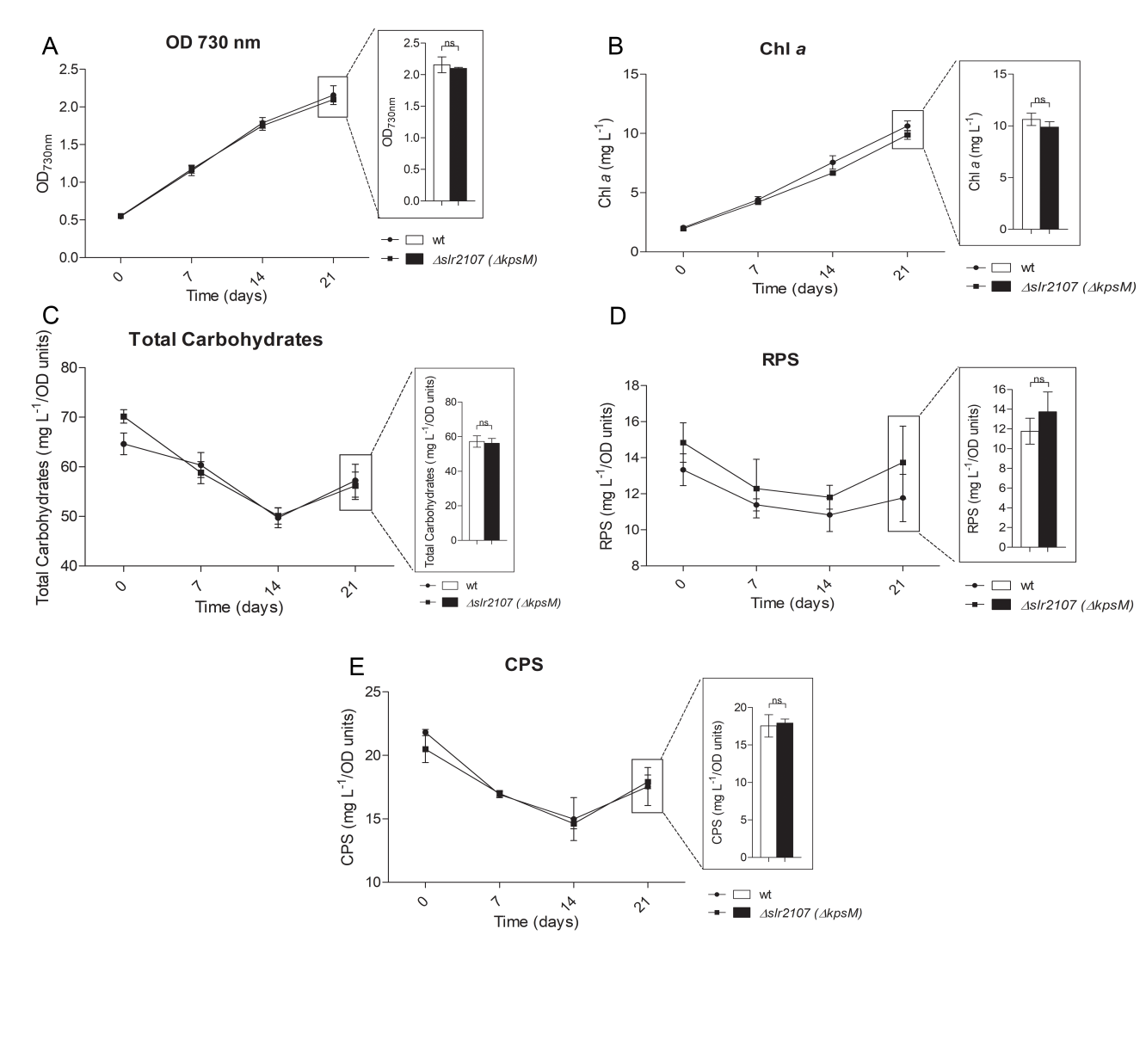
**

**Fig. S4.** Characterization of *Synechocystis* sp. PCC 6803 wild type and Δ*kpsM* mutant in terms of growth [(A) optical density at λ=730nm (OD_730nm_) and (B) µg of chlorophyll *a* per mL of culture (Chl *a*)], and production of (C) total carbohydrates, (D) released polysaccharides (RPS) and (E) capsular polysaccharides (CPS) expressed as mg per OD730_nm_ units. Cells were grown in BG11 medium at 30 ºC, under a 12 h light (50 µE m^-2^ s^-1^)/12 h dark regimen at 150 rpm. Experiments were performed in triplicate. Data are means ± SD. Statistical analysis performed using one-way analysis of variance (ANOVA), followed by Tukey's multiple comparisons is presented for the last time point. Ns: no significant differences.

**
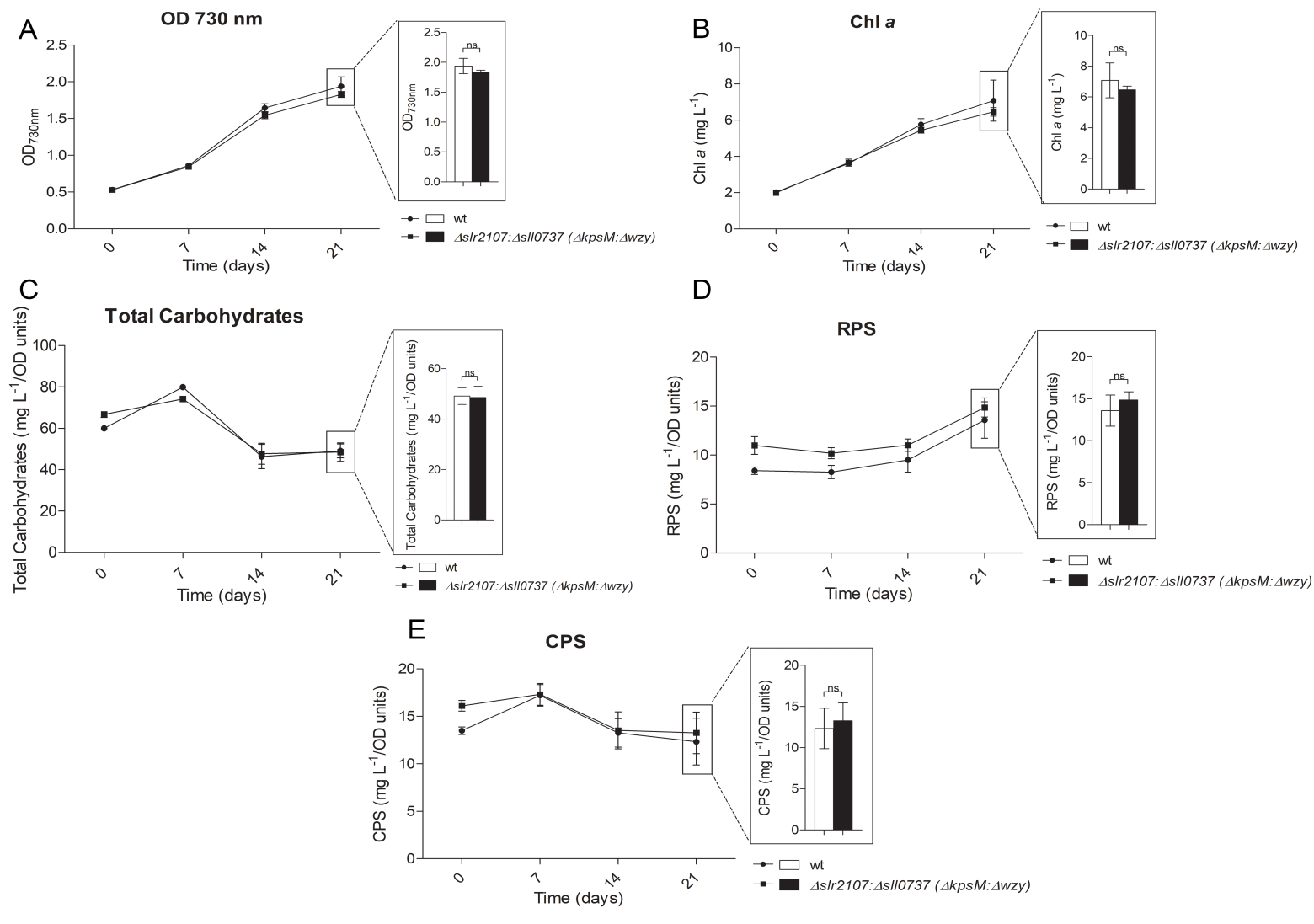
**

**Fig. S5.** Characterization of *Synechocystis* sp. PCC 6803 wild type and Δ*kpsM*Δ*wzy* mutant in terms of growth [(A) optical density at λ=730nm (OD_730nm_) and (B) µg of chlorophyll *a* per mL of culture (Chl *a*)], and production of (C) total carbohydrates, (D) released polysaccharides (RPS) and (E) capsular polysaccharides (CPS) expressed as mg per OD730_nm_ units. Cells were grown in BG11 medium at 30ºC, under a 12 h light (50 µE m^-2^ s^-1^)/12 h dark regimen at 150 rpm. Experiments were performed in triplicate. Data are means ± SD. Statistical analysis performed using one-way analysis of variance (ANOVA), followed by Tukey's multiple comparisons is presented for the last time point. Ns: no significant differences.

**Fig. S6.** Transcription profiles, evaluated by RT-PCR, of the putative *wzy* (A), *wzx* (B) and *kpsM* (C) gene copies in *Synechocystis* sp. PCC 6803 wild type (wt) and ∆*wzy* (*∆sll0737*), ∆*wzx* (*∆sll5049*) and ∆*kpsM* (*∆slr2107*) mutants, respectively. Samples for RNA extraction were collected 6h into the light period of the 12h light (50 μE m^-2^ s^-1^) / 12h dark growth regimen at 30^o^C. The cDNAs were produced with random primers and used in PCR amplifications with specific primer pairs. Expected size of PCR products: *wzy*: 1 – *sll0737* (465 bp); 2 – *slr0728* (288 bp); 3 – *slr1515* (256 bp); 4 – *sll5074* (440 bp); 5 – *slr1074* (368 bp); *wzx*: 1 – *sll5049* (223 bp); 2 – *slr0488* (274 bp); 3 – *slr0896* (323 bp); 4- *slr1543* (208 bp); *kpsM*: 1 – *slr2107* (203 bp); 2 – *slr0977* (220 bp); 3 – *sll0564* (396 bp). RT-PCR controls were performed using RNA from *Synechocystis* wild-type and mutants as template. Amplification of the housekeeping gene *rnpb* was used as positive control (D) (Pinto *et al.*, 2012).

**
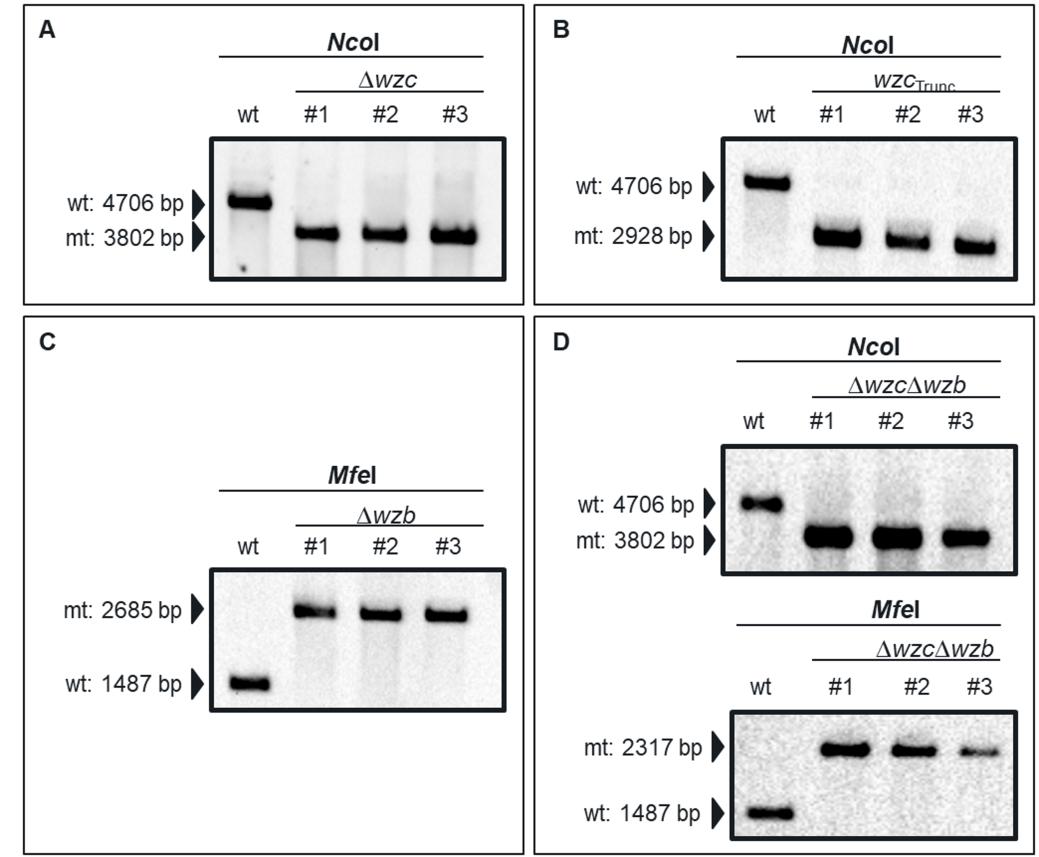
**

**Fig. S7.** Southern blot analysis confirming the segregation of the *Synechocystis* sp. PCC 6803 mutants (A) ∆*wzc*, (B) *wzc*_Trunc_*_,_* (C) ∆*wzb* and (D) ∆*wzc*∆*wzb* (∆*wzc* segregation – upper blot; ∆*wzb* segregation – lower blot). The DNAs were digested with the endonuclease indicated. A dioxigenin labeled probe covering the 5´ or 3’ flanking region of *wzb* or *wzc*, repectively was used. The sizes of the DNA fragments are indicated. wt – wild type; # clone tested.


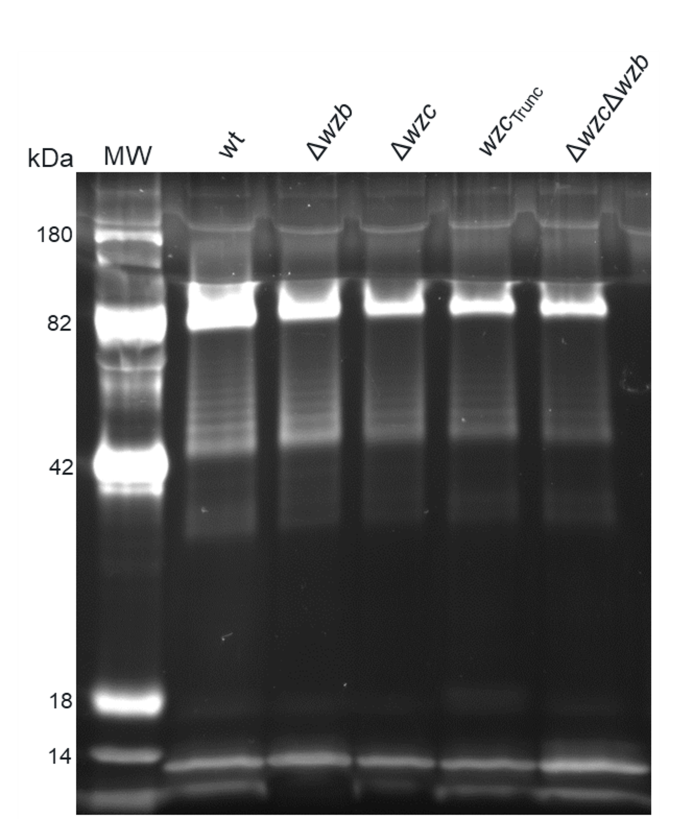


**Fig. S8.** Analysis of outer membrane preparations from *Synechocystis* sp. PCC 6803 wild type (wt) and the ∆*wzb*, ∆*wzc*, ∆*wzc*_trunc,_ and ∆*wzc*∆*wzb* mutants. Samples were resolved in Tris-glycine 12 % SDS gels and visualized using the Pro-Q® Emerald 300 Lipopolysaccharide Gel Stain Kit. LPSst: 0,5 µg of smooth LPS standard from *E. coli* serotype 055:B5 (control); MW: CandyCane™ glycoprotein molecular weight standards.


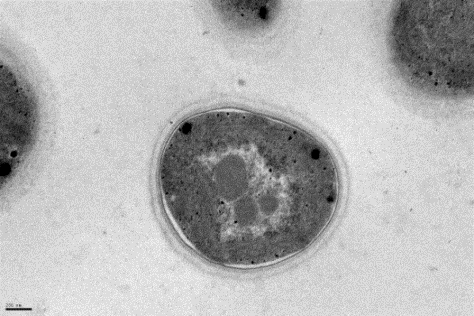

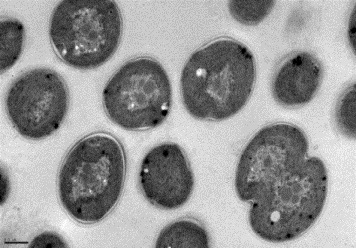


**D**


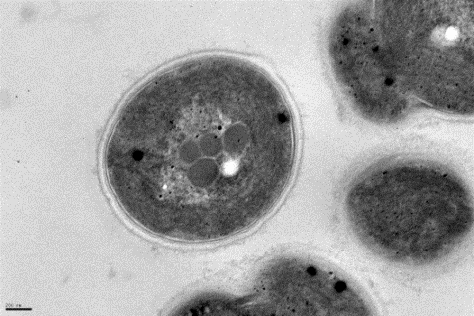

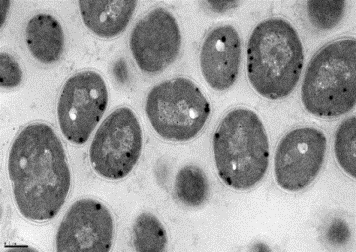


**B**


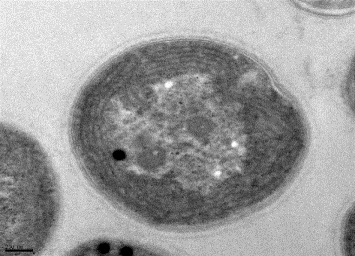

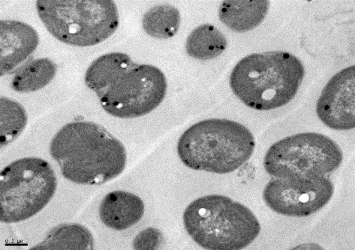


**C**


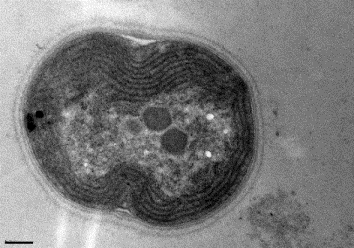

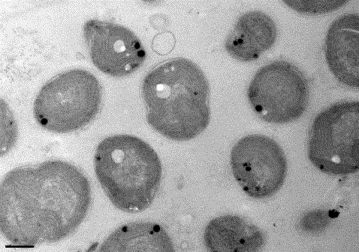


**E**


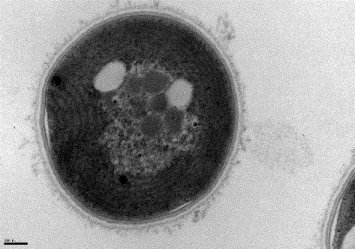

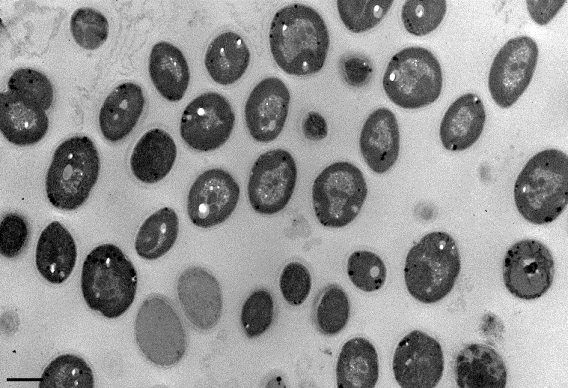


**A**


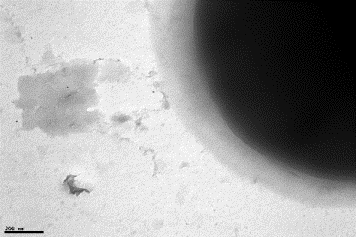

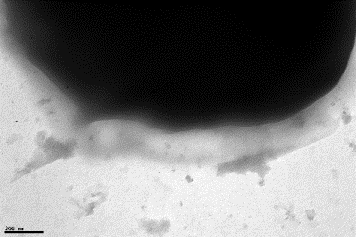

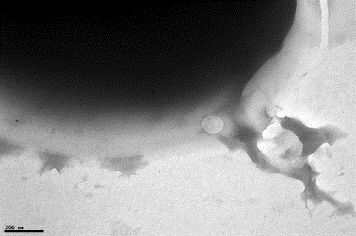

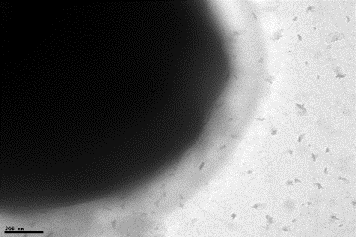

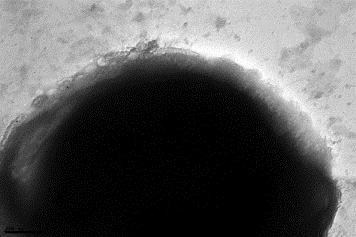


**Fig. S9.** Transmission electron micrographs of *Synechocystis* sp. PCC 6803 (A) wild type, (B) ∆*wzc*, (C) *wzc*_trunc_, (D) ∆*wzb*, and (E) ∆*wzc*∆*wzb*. Right panel – ruthenium red negatively stained cells. Size bars: left panel - 0.5 µm; middle and right panel - 0.2 µm.


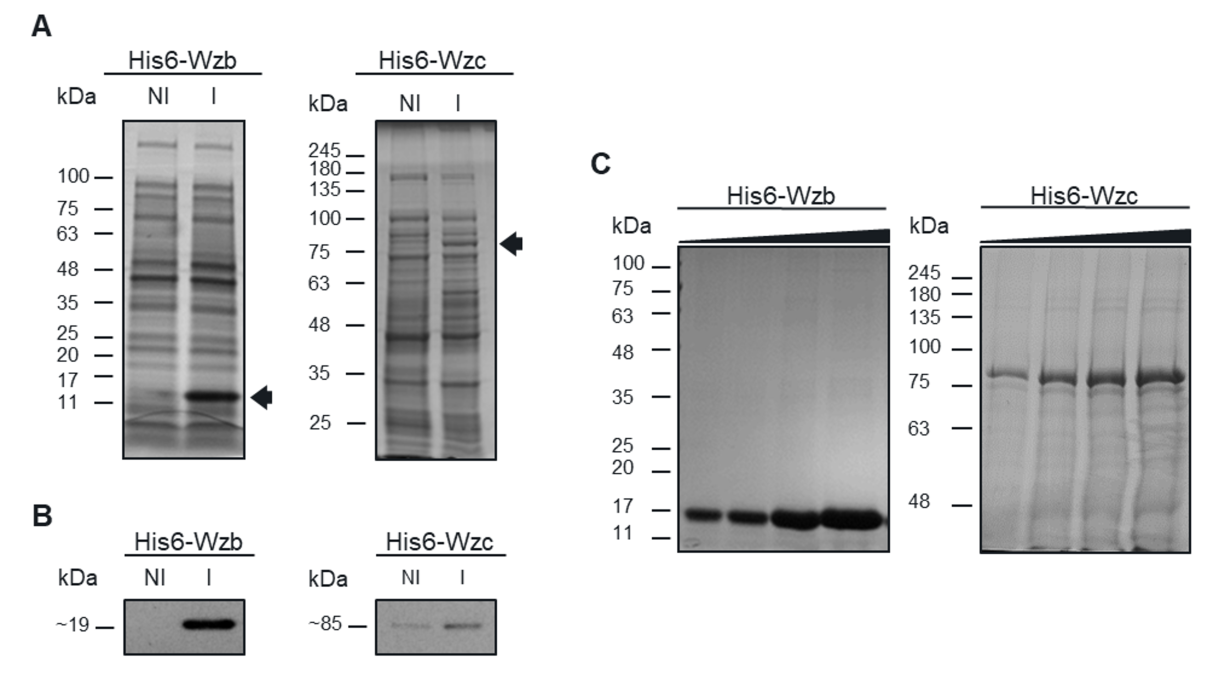


**Fig. S10.** Overexpression and purification of *Synechocystis* sp. PCC 6803 His6-Wzc and His6-Wzb. (A) SDS-PAGE analysis of crude cell extracts of *E. coli* M15(pREP) cells harboring plasmid pQE-30 encoding the His6-Wzc or His6-Wzb. Arrow heads indicate the target overexpressed protein. NI – non-induced cells, I – induced cells (IPTG). (B) Western blot analysis of His6-Wzc or His6-Wzb using a 6x-His epitope tag antibody. (C) SDS-Page analysis of increasing amounts of purified His6-Wzb (0.75, 1.5, 7.5 and 15 µg) and His6-Wzc (3.0, 6.0, 9.0 and 12.0 µg). Molecular mass standards are indicated on the left.


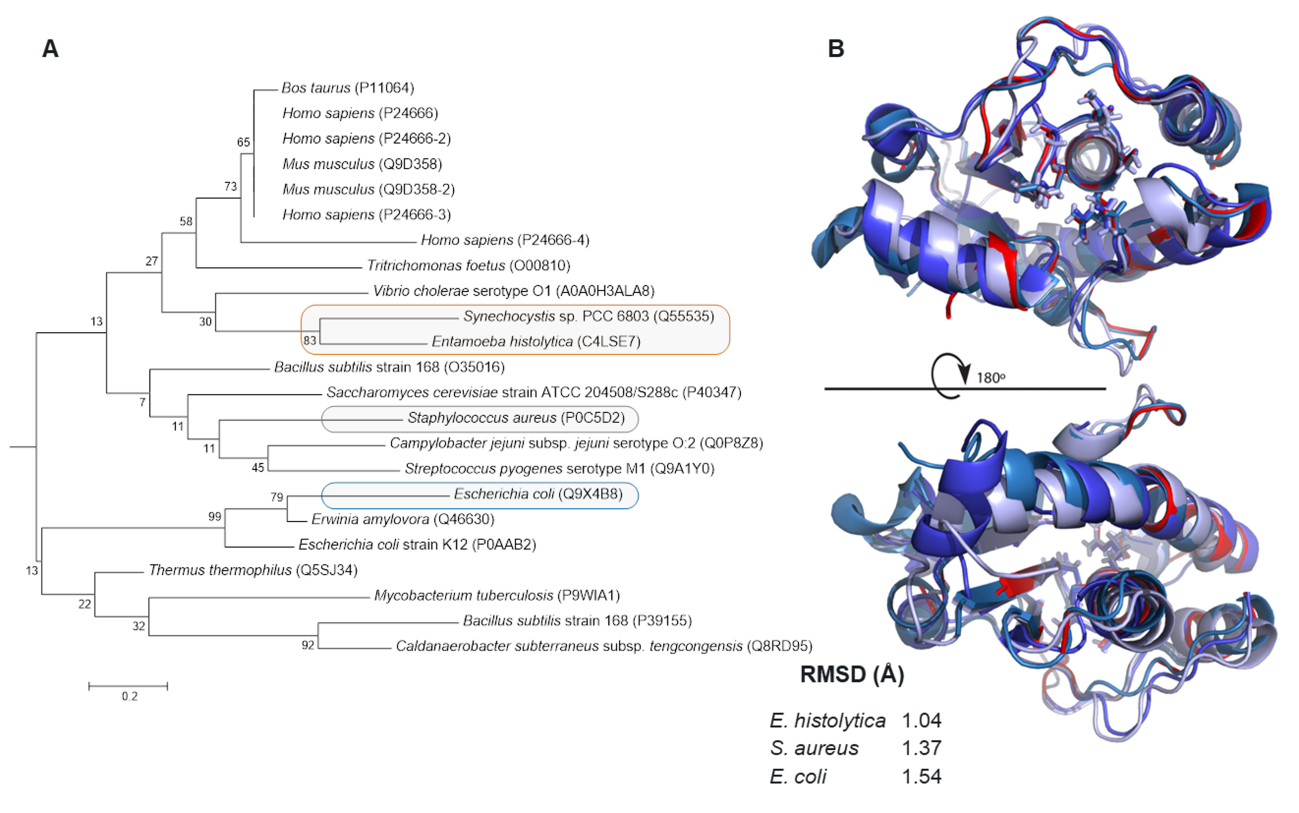


**Fig. S11.** Phylogenetical relationships and structural alignment and of *Synechocystis* sp. PCC 6803 Wzb. (A) Phylogenetic tree of Wzb and available homologs, as defined by Blast searches, with available crystal structure in PDB. Sequences are identified by the name of the organism and the UniProt entry within brackets. Sequences selected for the structural alignment are highlighted. (B) Structural alignment of Wzb (red) against LMW-PTP from *E. coli* (PDB: 2wja; UniProt: Q9X4B8; dark purple), *S. aureus* (PDB: 3rof; UniProt: P0C5D2; light purple) and *E. histolytica* (PDB: 3ido; UniProt:C4LSE7; blue). An overall structural similarity is visible, including active site superposition, as indicated by root mean square deviation (RMSD) values (Å): 1.54, 1.04 and 1.37 for Wzb against homolog from *E. coli*, *E. histolytica* and *S. aureus*, respectively (protein backbone in cartoon representation and catalytic residues in stick representation).


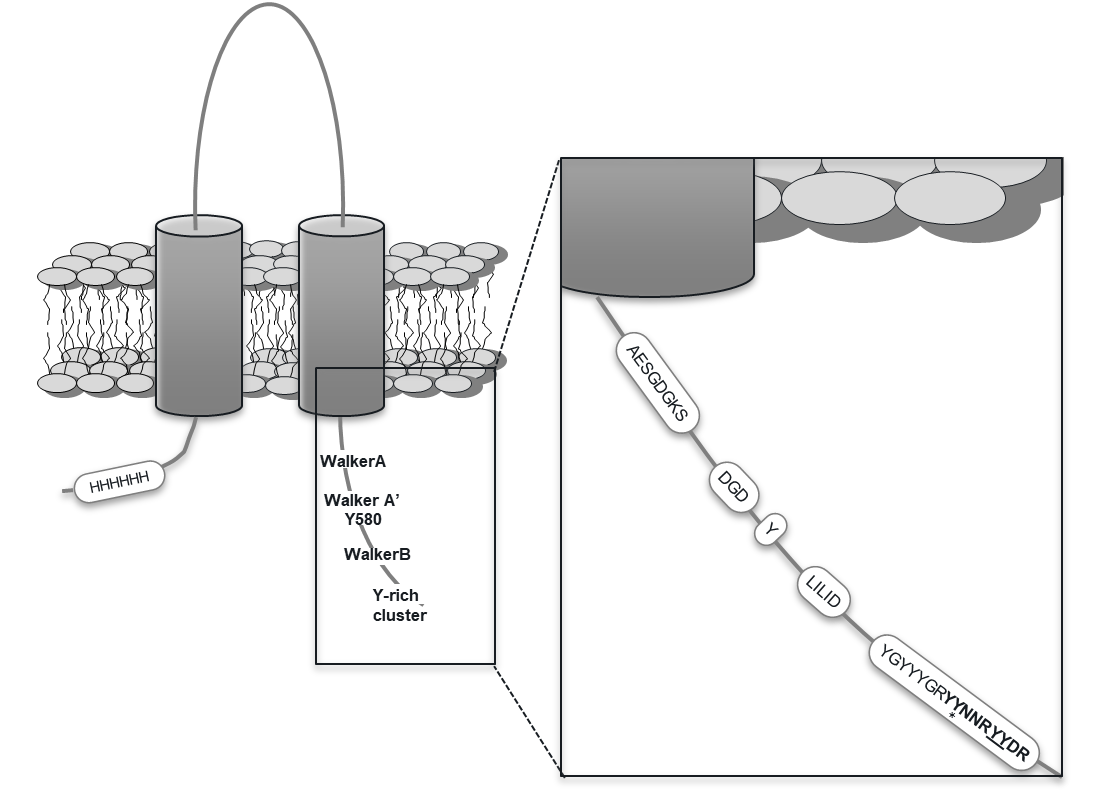


**Fig. S12.** Predicted topology of the Wzc protein according to the Phyre^2^ database. The His6-tag on the N-terminal sequence is depicted. The C-terminal citoplasmic domain contains a canonical Walker A, Walker A’, conserved tyrosine (Y) at position 580, Walker B and a C-terminal tyrosine rich cluster. The results from His6-Wzc MS/MS confirmed the phosphorylation of the C-terminal residues Y745 and Y746 (underlined). Residue Y741 (marked with an *) may also be phosphorylated, but further evidence is needed.

**References**

Jittawuttipoka, T., M. Planchon, O. Spalla, K. Benzerara, F. Guyot, C. Cassier-Chauvat & F. Chauvat, (2013) Multidisciplinary Evidences that *Synechocystis* PCC6803 Exopolysaccharides Operate in Cell Sedimentation and Protection against Salt and Metal Stresses. *PLoS One* **8**: e55564.

Pinto, F., C.C. Pacheco, D. Ferreira, P. Moradas-Ferreira & P. Tamagnini, (2012) Selection of suitable reference genes for RT-qPCR analyses in cyanobacteria. *PLoS One* **7**: e34983.

Pinto, F., C.C. Pacheco, P. Oliveira, A. Montagud, A. Landels, N. Couto, P.C. Wright, J.F. Urchueguía & P. Tamagnini, (2015) Improving a *Synechocystis*-based photoautotrophic chassis through systematic genome mapping and validation of neutral sites. *DNA Res.* **22**: 425-437.

Silva-Rocha, R., E. Martínez-García, B. Calles, M. Chavarría, A. Arce-Rodríguez, A. de las Heras, A.D. Páez-Espino, G. Durante-Rodríguez, J. Kim, P.I. Nikel, R. Platero & V. de Lorenzo, (2013) The Standard European Vector Architecture (SEVA): a coherent platform for the analysis and deployment of complex prokaryotic phenotypes. *Nucleic Acids Res.* **41**: D666-D675.
